# MECHANISMS OF TECOVIRIMAT ANTIVIRAL ACTIVITY AND POXVIRUS RESISTANCE

**DOI:** 10.21203/rs.3.rs-5002222/v1

**Published:** 2024-09-23

**Authors:** Riccardo Vernuccio, Alejandro Martínez León, Chetan S. Poojari, Julian Buchrieser, Christopher Selverian, Yakin Jaleta, Annalisa Meola, Florence Guivel-Benhassine, Françoise Porrot, Ahmed Haouz, Maelenn Chevreuil, Bertrand Raynal, Jason Mercer, Etienne Simon-Loriere, Kartik Chandran, Olivier Schwartz, Jochen S. Hub, Pablo Guardado-Calvo

**Affiliations:** 1 G5 Structural Biology of Infectious Diseases, Institut Pasteur, Université Paris Cité, Paris, France; 2 Theoretical Physics and Center for Biophysics, Saarland University, 66123, Saarbrücken, Germany; 3 Virus & Immunity Unit, Institut Pasteur, Université Paris Cité, CNRS, UMR 3569, Paris, France; 4 Department of Microbiology and Immunology, Albert Einstein College of Medicine, Bronx, NY, USA; 5 Cristallography Platform-C2RT, UMR3528, Institut Pasteur, CNRS, Université de Paris, Paris, France; 6 Plate-forme de Biophysique Moleculaire-C2RT, Institut Pasteur, CNRS UMR 3528, Université Paris Cité, Paris, France; 7 Institute of Microbiology and Infection, School of Biosciences, University of Birmingham, Birmingham, UK; 8 G5 Evolutionary Genomics of RNA Viruses, Institut Pasteur, Université Paris Cité, Paris, France

## Abstract

Mpox is a zoonotic disease endemic in central and west Africa. However, since 2022, human-adapted mpox virus (MPXV) strains are causing large outbreaks spreading outside these regions, leading the World Health Organization to declare public health emergency twice. Tecovirimat, the most widely used drug to treat these infections, blocks viral egress through a poorly understood mechanism. Tecovirimat-resistant strains, all with mutations in the viral phospholipase F13, pose public health concerns. Herein, we report the structure of an F13 homodimer, both alone and in complex with tecovirimat. We demonstrate that tecovirimat acts as a molecular glue, inducing the dimerization of the phospholipase. F13 escape mutations in MPXV clinical isolates are at the dimer interface and prevent drug-induced dimerization in solution and cells. These findings, which decipher tecovirimat’s mode of action, will allow better monitoring of poxvirus outbreaks and pave the way for developing more potent and resilient therapeutics.

## INTRODUCTION

Orthopoxviruses (OPXVs) cause various human diseases. Variola (VARV) and monkeypox (MPXV) viruses, are responsible for smallpox and mpox diseases, respectively. Other species, such as cowpox (CPXV), camelpox (CMLP), and borealpox (BRPV, formerly Alaskapox), trigger sporadic zoonotic infections^1^. Vaccinia virus (VACV), the prototype of the genus, has historically been used as a vaccine to eradicate smallpox. Smallpox vaccination was discontinued about 50 years ago and most of the population is immunologically naive to OPXV. This raises concerns about the emergence of zoonotic OPXVs or the impact of deliberate reintroduction of VARV as a bioweapon. Since 2022, two major mpox epidemics emerged. The first, caused by a low-virulence clade IIb strain, spread quickly across the globe, resulting in more than 90,000 cases and 179 deaths^2^. The second, produced by a more virulent clade Ib strain, produced the largest outbreak ever recorded in the Democratic Republic of Congo and spread to neighboring countries, resulting in thousands of deaths^3^. None of these epidemics has been resolved as of August 2024, and viruses continue to circulate in the population. First-generation vaccines do not meet modern safety standards, and attenuated third-generation vaccines, although effective, are difficult to produce on a large scale and do not induce long-term immunity ^4^. Most recently, mRNA-based candidate vaccines have demonstrated efficacy in animal models and are currently undergoing clinical trials^5,6^.

**Fig. 1.**
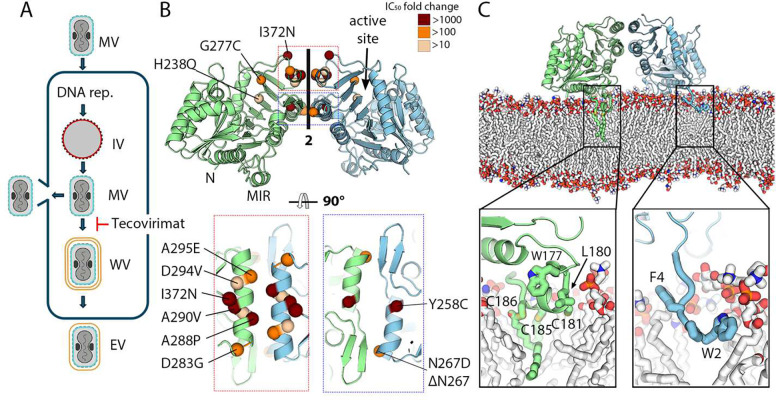
F13 forms a homodimer that can be inserted into a membranes surface. **(A)** Schematic representation of the replication cycle of OPXVs. Mature viruses (MV) enter cell fusing their membrane (in blue) with the cellular one. After DNA replication, immature particles are formed (IV, membrane in red), which give rise to intracellular MV particles in the cytoplasm of the infected cell. MVs can either be released by lysis or wrapping. In the latter, MVs acquire two additional membranes (in yellow) from the Golgi apparatus or endosomal vesicles to form wrapped virions (WVs), fuse the outermost with the plasma membrane and release enveloped viruses (EVs). Tecovirimat blocks wrapping, as indicated. **(B)** Crystal structure of the sF13 homodimer represented in cartoon. One protomer is colored blue and the other green. The N-termini and the membrane-interacting region (MIR) are indicated on one protomer, and the phospholipase active site is indicated on the other. Bottom panels provide close-up views of the two regions forming the dimer interface, indicated by colored rectangles in the upper panel. All single escape mutants identified to date are shown as spheres, colored according to their potency, reported as IC50 fold change. **(C)** Side view of the F13 homodimer interacting with a lipid membrane that mimics Golgi membrane composition, as observed from MD simulations. For clarity, water molecules and lipids in the foreground of the membrane are not shown. sF13 chains are colored as in (B), with palmitoylated cysteines and hydrophobic residues in the MIR and N-termini depicted as sticks. The bottom panel provides close-up views to show lipid-protein interactions, with the protein residues involved in the interaction depicted as sticks and labeled. Protein carbons are colored according to the chain, membrane carbons in white. Nitrogen, oxygen, sulphur, and phosphate atoms are colored blue, red, yellow, and orange, respectively.

OPXVs have an unusual replication cycle that produces two different viral particles termed mature (MV) and enveloped (EV) virions. MVs are produced in the cytoplasm and formed by a viral capsid wrapped in a single membrane. To disseminate within the host, MVs form wrapped virions covered by three membranes, which henceforth fuse the outermost with the plasma membrane to leave the host cell as enveloped viruses (EV), covered by two remaining membranes (Fig. 1A). Two oral drugs have been approved for the treatment of smallpox and mpox: brincidofovir (Tembexa) and tecovirimat (TPOXX). Brincidofovir is an inhibitor of viral DNA polymerase that has been shown to produce side effects in mpox patients^7^, and is mostly used to treat cases ineligible for tecovirimat. Tecovirimat blocks MV wrapping and has been widely used to treat mpox patients since the 2022 outbreak. However, it has a low resistance barrier, and multiple tecovirimat-resistant MPXV strains have been reported^8,9^. Resistance mapping studies^9^ have indicated that tecovirimat targets the major EV envelope protein F13, a membrane-anchored phospholipase that interacts with proteins involved in endosomal trafficking and plays a key role in the production of wrapped virions^10,11^. Tecovirimat binding to F13 blocks viral wrapping^12^ but the mechanism is poorly understood. Molecular dynamics (MD) simulations based on predicted structures have suggested different tecovirimat binding sites^13,14^, but none of them explain either how tecovirimat blocks wrapping or why known escape mutants escape the drug. This paucity of structural and mechanistic data prevents anticipating which mutations may confer resistance to tecovirimat and the development of better drugs.

Here, we elucidate using X-ray crystallography and molecular dynamics (MD) simulations the structure of F13 with or without tecovirimat. We showed that F13 forms a homodimer on membranes and tecovirimat inserts into a cavity formed between the two protomers. Using analytical ultracentrifugation (AUC), size-exclusion chromatography coupled with small-angle X-ray scattering (SEC-SAXS), mass photometry (MP), a proximity ligation assay (PLA), and binding free energy calculations we further report that tecovirimat induces homodimerization of F13 in solution and within cells, but not in resistant mutants, providing a mechanistic basis for drug activity and viral escape.

## RESULTS

### Tecovirimat binds a pocket between two protomers

F13 is a phospholipase anchored to membranes via two palmitoylated cysteines located in a hydrophobic membrane-interacting region (MIR, Fig. S1)^15^. For the structural studies, we produced a soluble variant of F13 (sF13) by removing the hydrophobic N-terminal tail (aa. 2–5) and by introducing five mutations in the MIR: W176A, F177A, C180A, C185A, and C186A. We obtained two different crystal forms (Table 1) and solved their structures by molecular replacement using a model generated by AlphaFold^16^. Similar to other phospholipase D (PLD) family members, sF13 has two repetitions of an α/β-hydrolase fold formed by a central β-sheet and two α-helical layers. In both crystal forms, sF13 featured a homodimer stabilized by two helices and a β-hairpin (Fig. 1B) with an interface area of 939 Å^2^ formed by a network of hydrogen bonds involving residues Y253, N259, N267, Y285, S292, and N300 (Fig. S2). The contact region forms a large cavity of 290 Å^3^. The hydrophobic N-termini and MIRs were located on one side of the dimer, and the two PLD catalytic pockets pointed outwards (Fig. 1B). To evaluate whether the dimer is compatible with membrane insertion, we performed molecular MD simulations on membranes mimicking the lipid composition of the Golgi membrane (Table 2). We observed that the homodimer remained intact during the 1,000 ns of simulation, suggesting that the dimeric interface is stable in the physiological membrane-bound state. sF13 was anchored to the membrane with the two palmitoylated cysteines inserted deeply into the outer leaflet of the membrane, while the N-terminal tail was associated with the lipid head group and glycerol regions (Fig. 1C). Two lines of evidence suggest that the homodimer has a biological role: (i) other membrane-interacting PLDs, such as the human exonucleases PLD3 and PLD4^17,18^, form similar homodimers with physiological roles; and (ii) mapping single escape mutants onto the sF13 homodimer reveals that all lie within or close to the dimer interface. Four escape mutations known to produce higher resistance to tecovirimat (Y258C, A288P, A290V, and I372N) were located at the center of the interface (Fig. 1B and Table S3). Moreover, an escape mutant (D280Y) of N1-isonicotinoly-N2–3-methyl-4-chlorobenzoylhydrazine (IMCBH), a selective inhibitor of vaccinia virus replication that blocks wrapped virion formation in vitro^19,20^, also mapped close to the dimer interface. These data indicate that F13 dimerizes when present on membranes and that this homodimer is targeted by tecovirimat and IMCBH.

**Fig. 2.**
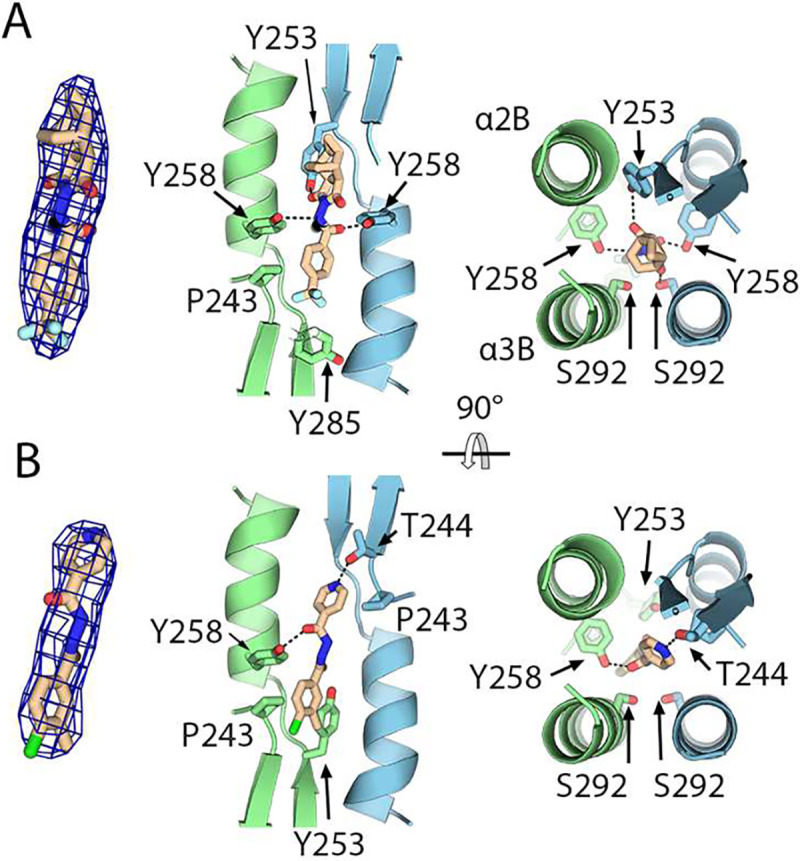
Tecovirimat binding site. **(A)** Crystal structure of the sF13/tecovirimat complex. The left panel shows a Fo-Fc omit map contoured at 3σ showing the density found at the dimer interface in the soaked crystal with the tecovirimat molecule modeled. The central and right panels provide orthogonal views of the dimerization interface with the tecovirimat molecule modeled and the residues contacting the drug represented as sticks and labeled. sF13 chains are colored as in Fig 1. **(B)** Crystal structure of the sF13/IMCBH complex. As in panel B, the left panel shows an omit map showing the electron density at the dimer interface in the soaked crystal, the central and right panels provide orthogonal views showing the sF13/IMCBH contacts.

To map the tecovirimat binding site at the atomic level, cubic crystals were soaked in a solution containing 1 mM tecovirimat and the structure was solved using molecular replacement. The resulting maps revealed an additional electron density at the dimeric interface compatible with the size and shape of a tecovirimat molecule (Fig. 2A). However, because the pocket is crossed by a crystallographic two-fold axis and tecovirimat is an asymmetric molecule, the resulting electron density is featureless, preventing the accurate modeling of the molecule using only the crystallographic density. To identify the correct atomistic pose and rationalize the featureless electron density, we performed absolute binding affinity calculations based on MD simulations of the F13 dimer and free energy perturbation (FEP) techniques. We first generated a set of 15 putative poses of different rotational states of tecovirimat that were compatible with the density. After equilibration of each pose by free MD simulations, we computed the absolute binding free energy (ABFE) for each initial pose using three independent sets of full FEP calculations. Because FEP achieves ABFEs with an accuracy of approximately 1–2 kcal/mol^21^, the calculations enabled us to identify the high-affinity poses selected by tecovirimat in the experiment. Notably, we identified multiple similar, yet clearly different poses with very low binding free energy values (ΔG_bind_) of less than −20 kcal/mol (Fig. S3 and Table S4). Averaging over 45 independent simulations (see Methods), we estimated an exceptionally strong affinity of ΔG_bind_ = −25.1 kcal/mol (95% confidence interval [−25.6; −23.5] kcal/mol), suggesting that tecovirimat strongly stabilizes the homodimer. In addition, the presence of multiple conformers with similarly high affinities is compatible with the presence of multiple tecovirimat conformers in the crystal, in agreement with the featureless electron density.

By refining the pose with the highest affinity against our crystallographic data, we observed that the molecule was stabilized by a network of polar contacts involving Y258 and S292 and hydrophobic contacts mediated by Y253, I262, I266, and Y285 (Fig. 2A). Similarly, we soaked sF13 cubic crystals with IMCBH and observed an electron density between both protomers that was compatible with the dimensions of IMCBH (Fig. 2B). Similar to tecovirimat, ICBHM is stabilized by a network of contacts involving Y258, S292, Y253, T244, and P243. Overall, the structural data revealed that both tecovirimat and IMCBH bind to the same pocket between the two protomers of the homodimer. The estimated ΔG_bind_ values suggest that tecovirimat stabilizes F13 homodimers, like a molecular glue.

### Tecovirimat stabilizes F13 homodimers

**Figure 3.**
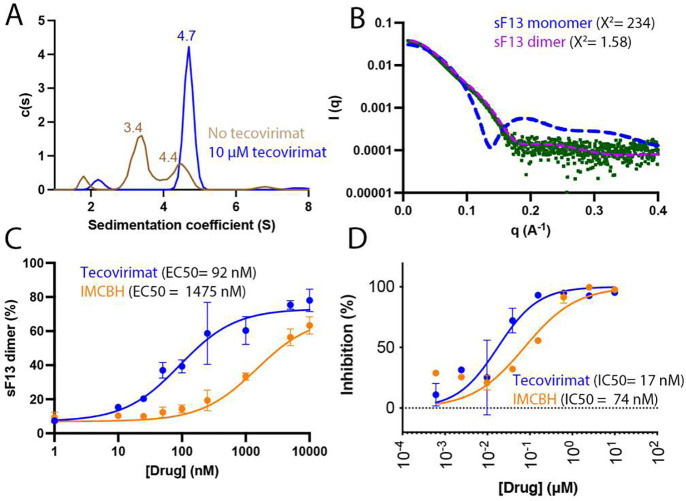
Tecovirimat induces sF13 dimerization in solution. **(A)** Analytical ultracentrifugation (AUC) analysis of sF13 without tecovirimat (brown line) and with 10 μM tecovirimat (black line). Experimentally derived sedimentation coefficient values (Svedberg units [S]) are shown above each peak. **(B)** Experimental SAXS profile (green dots) and theoretical profiles (dashed) calculated using CRYSOL ^79^ for one monomer of sF13 (blue line) and the dimer shown in Fig. 1 (pink line). **(C)** Dose-response curve used to estimate tecovirimat effect in solution. The Y-axis represents the proportion of dimers in a dilute solution of F13 measured by mass photometry (MP). The X-axis represents the concentration of drug (tecovirimat or IMCBH) present in the solution. The EC50 values were determined from a dose-response curve fitted using GraphPad Prism. **(D)** Tecovirimat (blue line) and IMCBH (orange) inhibits plaque formation of MPXV. Vero cells were infected with MPXV clade IIb and treated with the indicated concentrations of tecovirimat or IMCBH. Plaque inhibition is expressed as a percentage, normalized to control conditions. Data are presented as mean and standard deviation.

Next, we evaluated the oligomeric state of sF13 with and without tecovirimat using analytical ultracentrifugation (AUC). In the absence of the drug (Fig. 3A), sF13 was predominantly monomeric in solution (sedimentation coefficient, S = 3.4), with a minor presence of a higher S coefficient species (S = 4.4), which we interpreted as a dimer. Consistent with our hypothesis, tecovirimat shifted the equilibrium to the dimeric form (S = 4.7), with no monomeric protein detected. To confirm that the tecovirimat-induced dimer in solution corresponded to the dimer observed in the crystals, we analyzed the sF13/tecovirimat complex using SEC-SAXS (Fig. 3B and Fig. S3). The molecular weights derived from the Guinier analysis (Fig. S3A) showed no sample aggregation and the Kratky plot indicated a well-folded globular protein (Fig. S3B). In agreement with the AUC experiments, the F13/tecovirimat complex behaved as a monodisperse distribution of dimers in solution with an estimated molecular mass of approximately 76–84 kDa, a maximum distance (Dmax) of 108 Å and a radius of gyration (Rg) of 32.9 Å, matching the calculated values of the crystallographic dimer of 88.6 kDa, 117 Å and 32.3 Å, respectively. For further structural insights, we compared the experimental SAXS curve with the theoretical curves of the F13 monomer and dimer (Fig. 3B) and found that the dimer curve fitted nicely with the experimental curve. Overall, these results demonstrate that tecovirimat induces dimerization of sF13 in solution and that the resulting dimer adopts the same organization as the dimer observed in the crystal structure.

Next, we assessed the activity of tecovirimat in solution. To do this, we designed an assay to calculate the concentration of the drug required to induce dimerization of 50% of sF13 (EC50). We used mass photometry (MP), which measures the molecular mass from individual molecules by quantifying light scattering in a dilute solution. This technique enables a higher throughput than AUC or SEC-SAXS and allows for the evaluation of a full range of tecovirimat concentrations. We compared the EC50 values obtained with the MP assay —92 nM for tecovirimat and 1475 nM for IMCBH (Fig. 3C and S4) — to the IC50 values measured in cells infected with a recently isolated clade IIb MPXV strain^22^ —17 nM for tecovirimat and 74 nM for IMCBH (Fig. 3D). In both assays, tecovirimat was 5–16 times more active than IMCBH. Therefore, the dimerization-inducing activity measured in solution correlated with the antiviral activity of the drugs. The higher EC50 values obtained in solution can be explained by the different levels of entropy loss during dimerization. On the membrane surface, F13 diffused in only two translational and one rotational dimension. In solution, additional translational and rotational degrees of freedoms are lost upon dimerization, which explains why increased drug concentrations are required for dimerization.

To identify the pathway by which tecovirimat induces dimerization, whether it binds to the monomer and induces dimerization or binds to a pre-formed dimer, we computed the binding affinity of tecovirimat to F13 protomers. Using the tecovirimat conformation with the highest affinity to the dimer, we removed one protomer from the MD simulation system and computed the affinity to the remaining protomer. Averaging over 6 independent simulations (see Methods) we estimated a ΔG_bind_ = −5.7 kcal/mol (95% confidence interval [−6.1; − 4.4]) (Table S5). This corresponds to an EC50 value of approximately 68 μM, indicating a significantly weaker affinity as compared to binding to the dimer. These findings suggest that tecovirimat mostly binds to encounter complexes of two F13 protomers, which are then stabilized as dimers by the binding of tecovirimat at a pre-formed dimer interface.

### Tecovirimat escape mutants prevent F13 dimerization

**Figure 4.**
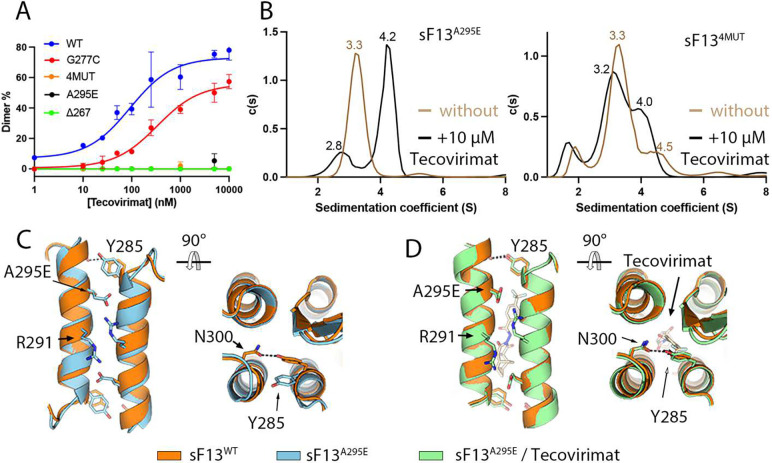
Escape mutants identified in mpox patients prevent tecovirimat-induced dimerization. **(A)** Mass-photometry-based dose-response curve showing tecovirimat activity against different escape mutants, as indicated. **(B)** Analytical ultracentrifugation (AUC) analysis of sF13^A295E^ (left panel) and sF13^4MUT^ (right panel) without tecovirimat (brown line) and with tecovirimat (black line). Experimentally derived sedimentation coefficient (S) values are shown above each peak. **(C)** and **(D)** are two orthogonal views showing the dimer interface of sF13^A295E^ (cyan, panel C) and sF13^A295E^/tecovirimat (green, panel D) superimposed on sF13^WT^ (orange). Residues E295, R291, Y285, and N300 are represented as sticks and labeled.

Taken together, these structural and functional data suggest that escape mutants may circumvent drug activity by preventing the formation of F13 homodimers. To test this hypothesis, we investigated the effect of escape mutants on tecovirimat activity using the MP assay. We tested three escape mutants identified in mpox patients treated with tecovirimat (Table 3): A295E, the quadruple mutant (4MUT) N267D, A288P, A290V, D294V, and ΔN267. We also assayed one escape mutant (G277C) identified upon in vitro passage of VACV, CPXV, and CMLV in the presence of the drug, but never reported in mpox patients. The mutations identified in clinical MPXV strains (N267D, ΔN267, A288P, A290V, D294V, and A295E) were located at the homodimerization interface and were expected to have an impact on tecovirimat-induced dimer formation, whereas G277C was located further away (Fig. 1B). Consistently, the MP assay showed that sF13^A295E^, sF13^4MUT^ and sF13^ΔN267^ did not form homodimers in the presence of tecovirimat at the range of concentrations tested, whereas sF13^G277C^ did as the wild-type protein (Fig. 4A). To complement the MP data, we repeated the AUC experiments reported above, which were performed with a much higher protein concentration, using sF13^A295E^ and sF13^4MUT^ (Fig. 4B and Fig. S5) and observed that the drug induced dimerization of sF13^A295E^ but not of sF13^4MUT^, which is in line with the observed ΔEC_50_ values.

Next, we aimed to elucidate the structural basis of the partial resistance of the A295E mutant. We obtained cubic crystals of sF13^A295E^ and soaked them in tecovirimat. In the absence of tecovirimat, sF13^A295E^ formed a homodimer that resembles that of sF13^WT^ but with the ends of a10 helix being slightly more open, so that Y285 was hydrogen bonded with Q299 instead of with N300, as in the wild-type protein (Fig. 4C). This results in a reduction in the buried surface area from 939 to 882 A^2^, likely reducing dimer stability. In the presence of tecovirimat, sF13^A295E^ recovered the native conformation in which Y285 was hydrogen bonded to N300 (Fig. 4D). We thought to extend this observation to another escape mutant (4MUT), but we failed to crystallize sF13^4MUT^. However, it is likely that the introduction of a proline in the middle of the a10 helix generates a major conformational reorganization that prevents F13 dimerization. Overall, we concluded that escape mutants isolated from tecovirimat-treated patients alter the dimerization interface of sF13, making tecovirimat-induced dimerization less efficient.

**Figure 5.**
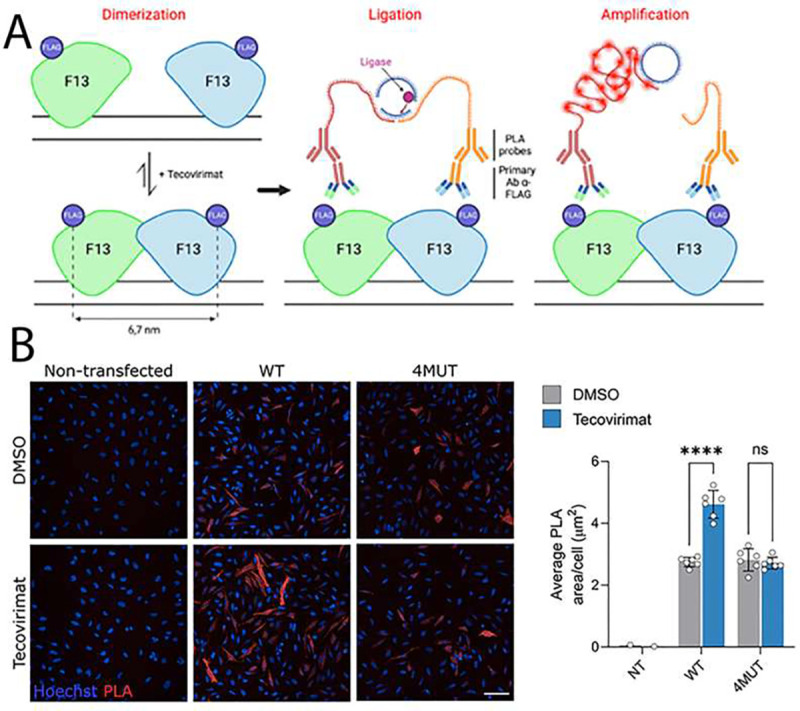
Tecovirimat induces F13 dimerization in cells. **(A)** Schematic model representing the PLA experiment. F13 protomers are colored green and cyan with the approximate location of the flag tag indicated with a blue sphere. The three steps of the assay: dimerization, ligation and amplification, are indicated. **(B)** The left panel are representative fluorescence microscopy images with the nuclei colored in blue and the PLA signal in red. Scale bars: 100 μm. The right panel is the quantification of the PLA signal as the average area of PLA fluorescence per cell. 7000 to 12000 cells were analyzed per data point. Data are mean±sd of two independent experiments performed in triplicat (n=6). Statistical analysis: Two-Way ANOVA. ns: non-significant, ****p < 0.0001.

We then investigated whether tecovirimat induced F13 dimerization in cells. To evaluate this, we used a proximity ligation assay (PLA). This technology is based on two oligonucleotide-labeled antibodies (PLA probes) that bind to the constant region of a pair of primary antibodies targeting the proteins of interest. If the probes are less than 40 nm apart, they hybridize and incorporate fluorescent oligonucleotides during the rolling-circle amplification that takes places, producing a fluorescent signal that can be visualized and quantified by fluorescence microscopy (Fig. 5A). Because we did not have specific antibodies targeting F13, we engineered a version of F13 with a flag tag in the loop connecting β1A to α1A, away from the dimerization and membrane interaction interfaces (Flag-F13^WT^). We also produced a variant that no longer interacted with tecovirimat (Flag-F13^4MUT^). We used two commercial antibodies targeting the flag tag, one with a mouse and the other with a rabbit Fc. Hela cells were transiently transfected with Flag-F13^WT^ or Flag-F13^4MUT^ and incubated with 10 μM tecovirimat or 0.1% DMSO as a control. PLA signal was quantified after 24 h of incubation. A signal was detected in F13-expressing cells but not in control cells. In the absence of tecovirimat, a signal was observed in cells expressing WT or mutant F13, likely corresponding to the basal levels of proteins that were less than 40 nm apart. Tecovirimat induced a significant increase in the PLA signal in cells expressing Flag-F13^WT^ but not in cells expressing Flag-F13^4MUT^ (Fig. 5B and S6). Therefore, we conclude that tecovirimat induces F13 dimerization in cells, and that escape mutants interfere with this dimerization.

### Dimer-stabilizing mutations render VACV non-viable

**Figure 6.**
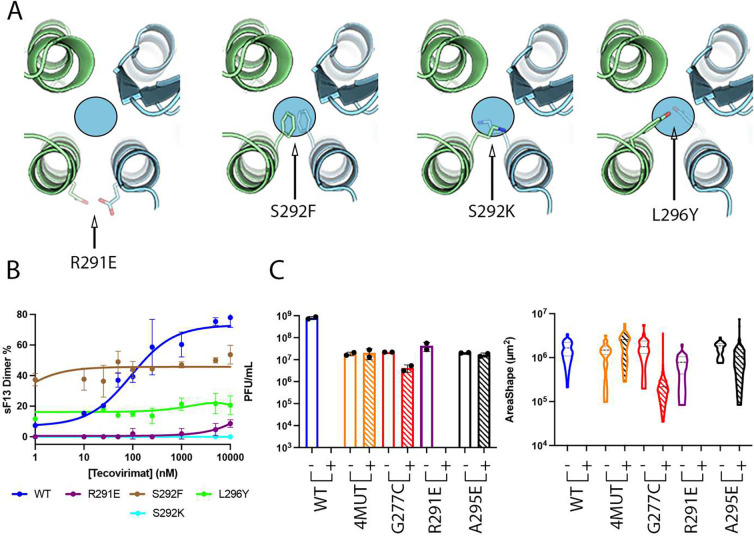
Structure-based escape mutations do not generate viable viruses. **(A)** Close view of the dimerization interface across the two-fold axis showing the designed mutations S292F, S292K and L296Y and the mutation identified in VARV, R291E. The circle indicates the localization of the tecovirimat-binding site. **(B)** MP-based dose-response curve showing tecovirimat activity against different mutants, as indicated. **(C)** Viral titers in PFU/mL (left panel) and plaque size (right panel) in the presence (+) and absence (−) of 10 μM tecovirimat calculated from plaque assays. Each bar represents the means ± standard deviation (SD) from replicate experiments. The limit of detection (LOD) is marked by the dashed horizontal line.

Isolation of tecovirimat-resistant MPXV strains revealed only ten mutations that, either isolated or in combination, confer resistance to the drug (Fig 1A and Table S2) ^8^. We wondered why other mutations did not emerge. One plausible explanation is that most mutations in this region are associated with a major loss of fitness, rendering the virus non-viable. To test this, we designed three escape mutants (S292F, S292K, and L296Y) by introducing bulky side chains into the tecovirimat binding cavity (Fig. 6A), and studied their sensitivity to tecovirimat in vitro using the MP assay. We confirmed that sF13^S292F^, sF13^S292K^ and sF13^L296Y^ were totally insensitive to tecovirimat activity (Fig. 6B). sF13^S292F^ and to lesser extent sF13^L296Y^ formed dimers in absence of the drug, suggesting that filling the cavity with hydrophobic side chains stabilizes the dimer. To assess viral viability, we introduced S292F into VACV using the MAVERICC system ^23^ to produce the recombinant virus rVACV^S292F^. As controls, we generated three additional viruses bearing substitutions in F13 known to confer tecovirimat resistance: rVACV^G277C^, rVACV^4MUT^, and rVACV^A295E^. As expected, all of the control viruses could be rescued and grew to high titers (Fig. 6C). However, despite multiple attempts, we were unable to generate rVACV^S292F^, suggesting that this substitution is deleterious to viral morphogenesis.

### Identification of unnoticed tecovirimat-escape mutants

We then sought for other tecovirimat resistance mutations that might have been unnoticed. To identify them, we extracted the F13 sequence from all MPXV genomes available on the GISAID database ^24^ , and from all OPXV available on the GenBank database. Within each species, we mapped variations in F13 amino-acid sequences in the dimer interface. No escape mutants were identified among the MPXV clade I sequences. However, we identified three potential escape mutants in MPXV clade II sequences: L296F, D280Y, and P243A, all from an immunocompromised patient treated with tecovirimat ^25^. Additionally, we identified R291E in one out of 81 analyzed VARV strains, the highly pathogenic strain India-1967. A structural analysis showed that R291E introduced two negatively charged residues facing each other on both sides of the interface (Fig. 6A), creating electrostatic repulsion that may hinder dimer formation and confer tecovirimat resistance. To test this, we first produced sF13^R291E^ and studied its sensitivity in vitro to tecovirimat. We showed that the drug has some activity at the highest concentrations in the assay (Fig. 6B). We next evaluated the resistance of rVACV^R291E^ to 10 μM tecovirimat by plaque assay (Fig. 6C). We used rVACV^WT^, rVACV^4MUT^, and rVACV^A295E^ as controls. As reported previously ^26^, tecovirimat abrogated plaque formation by rVACV^WT^. The control mutants completely (A295E, 4MUT) or partially (G277C) escaped tecovirimat, as determined by measurements of both plaque number and plaque area (Fig. 6C), providing evidence that F13 substitutions conferring tecovirimat resistance in MPXV are also transferable to VACV. However, the mutant R291E remained sensitive to tecovirimat, at least at the high drug concentration we used in these assays.

## Discussion

Here, we present the first structural study of viral phospholipase F13, including its interaction with tecovirimat. Previous studies have identified three important region for F13 activity: two palmitoylated cysteine residues (C185, C186) required for membrane association ^15^, a phospholipase motif (aa. 312–319) ^27–29^ and a di-aromatic motif (Y253, W254) required for interaction with late endosome proteins ^10^. Similar to tecovirimat binding, mutations in any of these residues reduce wrapped virion formation ^10,30^, but none of the escape mutants identified to date are in these regions ^8^. Our structural and MD data showed that F13 forms a homodimer on membranes, and structural comparison with other phospholipases revealed that this homodimer closely resembles that of phospholipases PLD3 and PLD4 ^18,31^, both transmembrane proteins with exonuclease activity. Sequence analysis showed that poxviral phospholipase K4, a paralog of F13 with nuclease activity ^32^, shares 48% sequence identity with human PLD3. Taken together, these findings suggest that poxviruses captured a PLD3-like gene from which both viral phospholipases evolved; K4 became a soluble protein but maintained its nuclease activity, whereas F13 remained in the membrane but acquired broad phospholipase activity ^29^.

Despite this evolutionary link, there is a significant difference between the homodimers of F13 and its eukaryotic counterparts: the presence of a large cavity in the dimerization interface of F13, which reduces dimer stability (Fig. S2). Sequence analysis revealed that this cavity is conserved across OPXVs, and most of the tecovirimat escape mutants are located around it. X-ray crystallography data and MD simulations showed that tecovirimat binds to the cavity with an exceptionally strong affinity, with some poses exhibiting binding free energies lower than −20 kcal/mol, demonstrating that tecovirimat serves as a highly potent molecular glue. Analytical ultracentrifugation, SEC-SAXS, mass photometry, and proximity-ligation assays confirmed that tecovirimat induced the dimerization of F13, both in solution and within cells.

Two main questions arise from these findings: (i) Why did evolution favor the formation of the cavity? and (ii) How does F13 dimerization blocks viral wrapping? We speculate that this interface has evolved to interact with viral and cellular partners, which explains its high degree of conservation. Supporting this hypothesis, Cheng et al. ^10^ showed that mutations in a di-aromatic motif located at the dimer interface (Y253A, W254A) prevent the interaction of F13 with the late endosome proteins Tip47/Rab9 and the formation of the wrapping complex. They also demonstrated that tecovirimat prevents the interaction of F13 with these proteins. These results suggest that tecovirimat-induced dimerization prevents F13 from interacting with its cellular partners, thereby blocking viral wrapping. In this line, we have shown that S292F, a mutation that stabilizes the dimer interface, renders the virus non-viable.

This model provides a molecular framework for understanding most escape mutants identified thus far: they alter the F13 dimerization region and prevent tecovirimat from inducing dimerization. To support this hypothesis, we developed a mass-photometry (MP) based assay to measure the activity of tecovirimat in solution and showed that A295E, 4MUT, and ΔN267 did not dimerize in the presence of the drug. The main limitation of the model is that it cannot explain why mutations such as G277C, which are not at the dimerization interface, escape the antiviral action of tecovirimat.

The results presented here have broad implications for public health. Estimating tecovirimat sensitivity of clinical isolates is important for monitoring epidemics. This is currently performed by isolating the virus and measuring its sensitivity to the drug in cell culture systems ^22,33^, which is labor-intensive and requires expensive and advanced equipment. The precise mapping of F13/tecovirimat contacts reported here provides the possibility of performing sequence-based estimation of tecovirimat sensitivity without the need to isolate the virus. Tecovirimat has demonstrated clinical efficacy in mpox patients, but no treatment alternatives are available for resistant strains. Our structural data, MD simulations, and the battery of assays developed here will pave the way for the development of novel antivirals active against tecovirimat-resistant mutants.

## MATERIALS AND METHODS

### Protein production and purification

To produce soluble F13 (sF13^WT^ and mutants), we cloned synthetic gene codon-optimized for expression in Escherichia coli into a pET-28a(+) vector (Novagen) with an N-terminal His- and Strep-tag followed by a thrombin site (GHHHHHHHHGSGAGWSHPQFEKGGSGLVPRGSGS). The sequence is derived from the Western Reserve strain of VACV (Uniprot code: P04021). We removed the hydrophobic N-terminal tail (residues 2–5) and introduced five mutations in the membrane interacting region (MIR): W177A, L178A, C181A, C185A and C186A, to remove the palmitoylation sites and the hydrophobic residues around. The point mutants mentioned in the text were introduced into this vector. We transformed E. coli BL21 (DE3) cells (New England Biolabs) and induced protein expression overnight at 16°C with 0.25 mM isopropyl β-d-1-thiogalactopyranoside (IPTG). We harvested cells from 3 L of culture and resuspended them in 40 mL cold resuspension buffer (Tris-HCl 10 mM pH 8, NaCl 150 mM, EDTA 1 mM) supplemented by one tablet of complete protease inhibitor (Roche), and frozen them at −20°C. The day after, we thawed and lysed them using a sonicator. After removing the insoluble material by centrifugating at 20,000 g (30 minutes, 4°C), we purified the recombinant protein using streptag based affinity chromatography in a StrepTrapTM HP 5 mL column (Cytiva), treated it with 5 mM Tris(2-carboxyethyl)phosphine hydrochloride (TCEP) (Thermo Scientific) for 10 minutes at room temperature to reduce all exposed cysteines, and purified the protein using size-exclusion chromatography with a Superdex 75 column (Cytiva) using SEC buffer (Tris-HCl 10 mM pH 8, NaCl 100 mM). The final yields obtained are: sF13^WT^ = 3.5 mg/L, sF13^A295E^ = 2.5 mg/L, sF13^G277C^ = 2.8 mg/L, sF13^4MUT^ = 1.4 mg/L, sF13^Δ267^ = 0.6 mg/L, sF13^R291E^ = 1 mg/L, sF13^S292F^ = 1.9 mg/L, sF13^S292K^ = 0.2 mg/L, sF13^L296Y^ = 0.4 mg/L. All proteins were analyzed by SDS-PAGE to assess their purity (Fig. S7).

### Crystallization and structure determination

For crystallization, we digested the purification tags using 1.5 units of thrombin (Cytiva) per 0.1 mg of protein overnight at 4°C and then treated the protein with TCEP for 10 minutes at room temperature. The digest was loaded to a gel filtration Superdex 75 16/60 column in 10 mM Tris HCl pH 8.0, 100 mM NaCl, and the fractions of the main peak were pooled and concentrated to 12 mg/mL in the same buffer for crystallization trials. Crystallization screening trials were carried out by the vapor diffusion method using a Mosquito TM nanodispensing system (STPLabtech, Melbourn, UK) following established protocols ^34^. Monoclinic crystals of sF13^WT^ grown after 10 days in 20% (w/v) PEG 3350, 0.1 M HEPES pH 7.5, 2% (v/v) Tacsimate and were cryoprotected in the same solution supplemented with 20% (v/v) glycerol. Cubic crystals of sF13^WT^ grown in 1 day in 1 M Na3 citrate, 0.1 M Imidazole pH 8, and were cryoprotected using the crystallization solution supplemented with 33% (v/v) glycerol. To obtain the complex with tecovirimat, we soaked cubic crystals, which have a high solvent content (70%), for 5 minutes into a soaking solution containing 1 mM tecovirimat (BenchChem, catalogue number: B611274), 10% (v/v) DMSO, 1 M Na3 citrate, 0.1 M Imidazole pH 8. To obtain the complex with N1-isonicotinoly-N2–3-methyl-4-chlorobenzoylhydrazine (IMCBH), we soaked cubic crystals into a solution containing 1 mM IMCBH (BLD Pharmatech Co., Ltd., catalogue number: BL3H9998EC8C), 10% (v/v) DMSO, 1 M Na3 citrate, 0.1 M Imidazole pH 8. After soaking, all crystals were cryoprotected using the soaking solution supplemented with 33% (v/v) glycerol. Similarly, cubic crystals of sF13^A295E^ were obtained in 1 day using 1 M Na3 citrate, 0.1 M Imidazole pH 8 and soaked with tecovirimat as reported above.

X-ray diffraction data were collected on beamlines PROXIMA-1 and PROXIMA-2A at the synchrotron SOLEIL (St Aubin, France). Diffraction images were integrated with XDS ^35^ and crystallographic calculations were carried out with programs from the CCP4 program suite ^36^. To determine the phases, we used a model of F13 obtained using AlphaFold2 ^16^ as a template to perform molecular replacement in PHASER ^37^. To obtain the final models, we iteratively built and refined the structures using phenix.refine ^38^ and coot ^39^ using isotropic B factor and TLS groups as refinement strategy. We validated all the models using Molprobity ^40^. The crystallographic statistics are provided in Table S1. The monoclinic crystals of sF13^WT^, which contained two protomers in the asymmetric unit, showed clear electron density for most residues except for the MIR (aa. 174–191), which could not be modeled. The geometry of the final model was good, with only four Ramachandran outliers: G119 and D280 in both chains. Cubic crystals of sF13^WT^ displayed one monomer in the asymmetric unit that showed clear density for the entire chain except for the MIR (aa. 176–185), where the density was weaker but clear enough to be modeled. The final model displayed good geometry with only one Ramachandran outlier. Crystals of sF13^WT^ soaked with tecovirimat or IMCBH showed clear electron density for the entire chain except for the region 176–186, where the density was weaker but sufficient to be modeled. Cubic crystals of sF13^A295E^ showed good electron density for the entire chain except for the MIR (aa. 175–185) and residues 280–282, where the density was weaker than in the crystals of sF13^WT^ because the A295E mutation induces flexibility in this region. However, this region could be modeled using a sigma cutoff of 1.1. The final model showed good geometry with only one Ramachandran outlier: D248. The structure of sF13^A295E^ soaked with tecovirimat showed clear electron density for the entire chain except for the MIR. The final geometry of the model was good with two Ramachandran outliers: G119 and P255. Cubic crystals of sF13^G277C^ showed good density for the entire chain except residues 176–188. The final model had good geometry and no Ramachandran outliers. In crystals soaked with tecovirimat or IMCBH, additional electron density appeared at the dimeric interface that was compatible with the shape and size of the drug, as shown in Fig. 2. To facilitate the modeling, all maps derived from the cubic crystals were corrected using a bulk-solvent mosaic model available in the PHENIX program (phenix.mosaic). Coordinates and structure factors have been deposited in the Protein Data Bank under the accession codes 9FHK, 9FHS, 9FI7, 9FJ1, 9FIZ, 9FJA and 9FJ0. Figures showing the crystallographic models were generated with PyMol (Schrödinger, LLC)

### Molecular dynamics simulations

To assess the stability of the X-ray resolved F13 dimer on the membrane surface, we conducted MD simulations. Prior to simulation, we made several structural modifications to the sF13 dimer. Firstly, the unresolved N-terminal residues (residues 1–5) were modeled as unstructured and integrated into the dimer structure using the Modeller tool ^41^. Next, the structure was processed using the CHARMM-GUI ^42^ server to add post-translational palmitoylation on residues Cys 185/186, and neutral capping of the N- and C-terminal residues. Subsequently, the F13 dimer was positioned on a membrane (Fig. 1C) mimicking the lipid composition of the Golgi membrane (as outlined in Table S2). For the protein force field, we used the CHARMM36m-WYF force field ^43,44^, which includes corrections for cation-pi interactions, while lipids were described using the CHARMM36 force field ^45^. The protein-membrane system was solvated using CHARMM-modified TIP3P ^46^ water molecules, and the total charge of the system was neutralized by adding 82 K+ ions. The total system size is 249,131 atoms, including 158,403 water molecules. The box dimensions were 14.34 × 14.34 × 13.56 nm in the x, y, and z directions. The solvated system was energy minimized using the steepest descent algorithm to remove any steric clashes, followed by six short equilibrations ranging from 125 ps to 500 ps with restraints on either protein backbone/side chain atoms or lipid phosphate atoms.

Throughout the equilibration process, we maintained a temperature of 310 K using the Berendsen thermostat ^47^ with a time constant (*τ*_*t*_) of 1 ps, while pressure was maintained at 1 bar using the Berendsen semi-isotropic scheme with a time constant (*τ*_*p*_) of 5 ps. van der Waals and electrostatic interactions were treated using the cut-off and Particle Mesh Ewald (PME) ^48,49^ methods, respectively, with a cutoff of 1.2 nm. H-bonds were constrained using the LINCS algorithm ^50^. For the final production run, we removed all restraints and switched to V-rescale ^51^ and Parrinello-Rahman ^52,53^ semi-isotropic scheme to regulate the temperature and pressure, respectively. The rest of the parameters were consistent with those used during equilibration. The production simulations were conducted for 1 microsecond with 5 repeats using the GROMACS 2021 simulation package ^54^, employing a time step of 2 fs. For analysis, we concatenated the last 300 ns from each repeat and examined monomer-monomer contacts within 5 Å. All images and plots were generated using VMD ^55^ and the matplotlib ^56^ library.

Although the structure of the sF13 dimer was determined at a resolution of 2.8 Å, a crystallographic 2-fold axis passes through the ligand density (Tecovirimat), leading to challenges in accurately fitting the ligand. To enhance the accuracy of ligand modeling within the density, we utilized the recently developed RosettaEMERALD ^57^ protocol. This protocol integrates both RosettaGenFF and genetic algorithm (GA) optimization for robust ligand modeling within the density map. The 3D structure of tecovirimat was downloaded from PubChem ^58^ in its endo-isomeric form, which is the favored product of the Diels-Alder reaction used to synthesize the drug. Next, the sF13 dimer-tecovirimat complex was docked to the density using the ChimeraX tool ^59^. Following this, we employed the RosettaEMERALD protocol to accurately model tecovirimat within the density. Briefly, an initial pool of 500 ligand conformations, along with protein side chains, undergo GA optimization over 10 generations. The top 20 lowest energy conformations obtained from GA optimization were further refined, along with protein side chains, using a cartesian minimization in Rosetta. The protocol was executed in triplicate. Out of the total 60 ligand conformations, redundant poses were eliminated, and 15 poses were selected for binding free energy calculations. These 15 selected poses are indicated by the first number of x-axis labels of Fig. S3. The RosettaEMERALD XML script and flags used for refining the ligand within the density are provided in the supplementary material.

### Absolute Binding Free Energy (ABFE) Calculation

The selected 15 poses were subjected to binding free energy estimation using an in-house pipeline (publication in preparation), detailed in Fig. S10 Our absolute binding free energy (ABFE) protocol is similar to the one previously described by Alibay et al. ^60^ ABFE calculations were performed in triplicate for each pose. To optimize the absolute binding free energy calculations, the membrane was excluded from the simulation. This simplification is justified as the tecovirimat binding pocket is located at 40 Å from the membrane surface, and equilibration simulations demonstrate that the interface remains stable throughout the simulation (Fig. S8). The AMBER-ff14sb ^61^ parameters for F13 dimer were acquired through the utilization of OpenMM ^62^ and ParmEd ^63^ software, while the OpenFF-2.0.0 ^64^ parameters with AM1- BCC charges for tecovirimat were obtained via TOFF ^65^ software. The TIP3P ^66^ water model was employed, along with AMBER parameters for ions. GROMACS-2022.4 ^54^ simulation package was used as the molecular dynamic engine.

In all cases, the simulation temperature was maintained at 298.15 K using Langevin dynamics with a collision frequency of 2 ps-1. Van der Waals and electrostatic interactions were treated using the cutoff and Particle Mesh Ewald (PME) methods ^48,49^, respectively, with a cutoff of 1 nm. Hydrogen bonds were constrained using the LINCS algorithm ^50^. Two different isotropic schemes were used to maintain the pressure at 1 atmosphere: Berendsen ^47^ with a time constant of 1 ps and Parrinello-Rahman ^52,53^ with a time constant of 2 ps. All production simulations used the former. We used a hydrogen mass repartitioning factor of 2.5, which allowed an integration time step of 4 fs for all production simulations. Other molecular dynamics parameters are detailed in the GROMACS input files provided as Supporting Information.

Our in-house pipeline operates as follow (see Figure S10): During the “Build Simulation System” phase, the user provides configuration details for the protein-ligand complex, and topologies are generated accordingly. Two neutral solvated systems with 150 mM of NaCl are created for the ligand and protein-ligand complex in an octahedron box with 1.5 nm distance between the solute and the edges’ box with the GROMACS’ solvate module. The “Equilibration Setup” and “Equilibration Run” steps generate molecular dynamics parameters and conduct the corresponding equilibration simulations. For the protein-ligand complex, the process begins with minimization using the steepest-descent algorithm. This is followed by a 1 ns NVT phase with a 2 fs integration time step and position restraints on the heavy atoms using a force constant of 2500 kJ mol−1 nm−2. Next, a 1.05 ns NVT phase and a 1.05 ns are conducted, both with a 3 fs integration time step and the same position restraints. The previous NPT phase utilizes the Berendsen scheme as detailed above. Subsequently, a 5 ns NPT phase with the Parinello-Rahman scheme and a 4 fs integration time step are performed without restraints. This is followed by a final step of 10 ns under the same conditions. For the ligand alone, the same procedure is followed, except the initial 1 ns NVT phase with a 2 fs integration time step is omitted. The final 10 ns of the simulation were used to estimate the optimal Boresch restraints for the decoupling phase of the protein-ligand complex simulations.

Similar to the previous two steps of the workflow, “FEP Setup” and “FEP Run” prepare and execute the free energy perturbation simulations needed to complete the thermodynamic cycle detailed in the Thermodynamic Cycle for Free Energy Perturbation (FEP) section. Each window for both the protein-ligand complex and the ligand alone begins with minimization using the steepest-descent algorithm. This is followed by a 10 ps NVT phase with a 2 fs integration time step and position restraints on the heavy atoms using a force constant of 2500 kJ mol^−1^ nm^−2^. Next, a 100 ps NPT phase is conducted with a 4 fs integration time step, the same position restraints, and the Berendsen scheme as detailed above. Subsequently, a 500 ps NPT phase with the Parrinello-Rahman scheme and a 4 fs integration time step is performed without restraints. This is followed by a final step of 10 ns under the same conditions.

The free energy contributions of each step are computed using either the multistate Bennett acceptance ratio (MBAR) ^67^ or Thermodynamic Integration (TI) estimators during the “Get Contribution” step. The Python package alchemlyb-2.0.0 ^68^ was utilized for this purpose. Finally, all results are aggregated in the “Get Cycle’s ΔG” step.

### Thermodynamic Cycle for Free Energy Perturbation (FEP)

The thermodynamic cycle involved decoupling the Coulomb interactions of the ligand in water over 11 λ points, followed by the decoupling of van der Waals interactions over 21 λ points with a soft-core potential activated to prevent numerical instability. Boresch restraints ^69^, chosen from the last 10 ns of the protein-ligand complex free simulation during the equilibration phase (see Absolute Binding Free Energy (ABFE) Calculation section), had their free energy contribution analytically calculated.

Both the selection and energy contribution estimation of Boresch restraints were conducted using the software MDRestraintsGenerator ^70^. The selected restraints were activated for the ligand in complex with the protein, and the van der Waals interactions of the ligand was reactivated in the protein complex over 21 λ points with a soft-core potential to avoid numerical instability. Subsequently, Coulomb interactions were activated over 11 λ points to finally remove the restraints over 12 λ points. The binding free energy is calculated from the contributions of all previously mentioned steps.

### Clustering and Identification of the Most Favorable Energetic Pose

Frames selected by MDRestraintsGenerator and used as input structures for the FEP simulations were clustered based on the protein-ligand interaction fingerprint (PLIF) calculated with ProLIF ^71^. Each bit of the fingerprint represents a pair of atom/atom groups from the protein and ligand involved in a specific class of interaction as defined by ProLIF. This unambiguous definition allows for the separation of potential poses that may be symmetrical. The final fingerprint consists of 956 bits. The similarity of each frame to the others is calculated using the Tanimoto metric implemented in RDKit ^72^ on the constructed protein-ligand interaction fingerprint, resulting in the generation of a similarity matrix.

The similarity matrix was then subjected to the hierarchical clustering algorithm in SciPy using Ward’s variance minimization algorithm ^73^. After constructing the dendrogram, the number of clusters was determined through visual inspection.

### ABFE between the most favorable energetic pose and the individual monomers

To investigate if tecovirimat can bind to the monomer, the ABFE for each individual monomer was calculated for the identified most favorable energetic pose within the dimer. The same methodology previously described was used, with the only difference being that a single monomer was used instead of the dimer.

### Calculation of Average Binding Free Energies

To estimate the average binding free energy for the dimer and monomer complexes with tecovirimat, we averaged over all independent simulations (*N*=45 for the tecovirimat–dimer, *N*=6 for the tecovirimat–monomer binding) as shown in Eq. 1, implying that we average with respect to the binding probabilities (rather than with respect to the binding free energies). Thereby, the average is dominated by the high-affinity binding poses. Here, β is the inverse temperature, and 〈·〉 denotes the average over independent simulations.

(1) $$\Delta G_{bind}=-\beta^{-1}ln\left\langle e^{-\beta \Delta G_{bind,i}}\right\rangle$$

The 95% confident interval was estimated from 1000 rounds of bootstrapping. In each round, $N\;\Delta G_{{\text{bind}}_{i}}$ samples were drawn with replacement from our $N\;\Delta G_{{\text{bind}}_{i}}$ values and averaged according to Eq. 1. After removing the largest and smallest 2.5% of the 1000 bootstrapped averages, 95% confidence intervals were obtained from the upper and lower bound of the remaining 950 averages.

### Mass Photometry (MP)

Mass photometry experiments were done using TwoMP instrument (Refeyn Ltd, Oxford, UK) using filtered (0.22 μm) “protein buffer” (10 mM Tris-HCl pH = 8, 100 mM NaCl) to avoid contaminations which would increase the background signal. Contrast-to-mass calibrations was achieved by measuring the contrast of two references (BSA and urease, both purchase from Sigma Aldrich) diluted in protein buffer, covering mass range from 66 kDa to 272 kDa. Four contrast values were used to generate a standard calibration curve, with following rounded average masses: 66, 132, 198, 272 kDa. We performed the experiment using microscope coverslips (24×50 mm and 170 ± 5 μm thick) cleaned with isopropanol and Milli-Q water followed by drying with air. Samples were loaded into dried coverslip surface assembled into silicone gaskets. Immediately prior to mass photometry measurements, 2 μL of sF13 protein stocks, with increasing amounts of tecovirimat or ICBMH, were diluted in 18 μL of “protein buffer” and deposited into the gasket hole. In all cases, the final concentration of sF13 was 25 nM. Tecovirimat/ICBMH were at different concentrations between 10 μM and 1 nM, as indicated in Figs. 3C, 6B and S5. Data acquisition was performed using either AcquireMP (Refeyn Ltd) and movies of 2936 frames were recorded at 49 Hz framerate, adjusted to maximize camera counts while avoiding saturation. MP images were processed and analyzed using DiscoverMP (Refeyn Ltd). Data processing consist in different steps, 1) background removal, 2) identification of landing particles on the glass surface and 3) particle fitting to extract the maximum contrast. Background removal is done by calculating ratiometric images, to reveal only the features that change between the two frame batches. This procedure is applied to all frames, resulting in a ratiometric movies. Landing particles on the glass surface generate a gradual increase in the glass reflectivity that produces an increase in the scattering signal, which is used to identify particles. Finally, to extract the contrast, identified particles are fitted using a PSF model.

Contrast-to-mass calibration was achieved analyzing with DiscoverMP (Refeyn Ltd) each calibration experiment. Mean contrast values from the BSA and urease calibration were plotted and fitted to a linear function y=bx, where y is the contrast, x is the mass and b is the contrast-to-mass calibration factor. To extract mole fractions (percentage of each species), we converted all particle contrasts obtained from each movie to mass, applied a gaussian fitting and calculated mole fractions as the area of each Gaussian curve. Finally, sF13 dimer percentage values were plotted against tecovirimat/ICBMH concentration using Prism Graphpad and EC50 values extracted using a nonlinear fit function (Fig. S5)

### Analytical ultracentrifugation (AUC)

Sedimentation velocity experiments were carried out at 20°C in an Optima AUC analytical ultracentrifuge (Beckman Coulter, Brea, CA, USA) equipped with double-UV and Rayleigh interference detection. Purified sF13 proteins at 0.4 mg/mL in presence or absence of tecovirimat (10 μM) were centrifuged at 42000 rpm using an AN60-Ti rotor and 12 mm thick double sector centerpieces. Absorbance and interference profiles were recorded every 5 min. Buffer viscosity (η= 1.016 cP) and density (ρ = 1.0054 g/mL) at 20 °C were estimated with SEDNTERP 1.09. Partial specific volumes at 20 °C were estimated based on amino acid sequences using SEDNTERP 1.09 software. Data were analyzed with SEDFIT 16.1 ^74^ using a continuous size distribution c(S) model. Theoretical sedimentations of the complex were generated using hydropro 10 ^75^.

### Small-angle X-ray scattering (SAXS) experiments

Small-angle X-ray scattering (SAXS) data were collected on the SWING beamline at Synchrotron Soleil (France) using the online HPLC system. These experiments have been performed using sF13^WT^ digested with thrombin. sF13^WT^ samples at 4.6 mg/mL were prepared in a buffer containing 10 mM Tris pH = pH 8, 100 mM NaCl, and 10 μM tecovirimat and injected into a size exclusion column (Superdex 75 increase 5/150mm) cooled at 15 °C eluting directly into the SAXS flow-through capillary cell at a flow rate of 200 μL/min. The data were analyzed using FOXTROT and PRIMUS from ATSAS 3.2 ^76^, from which Guinier was generated. Scattering curves were selected for stable radius of gyration (Rg) at the apex of the elution profile, and the selected curves were averaged, and buffer signal was subtracted. From these corrected scattering curves, the pair distribution functions was computed using GNOM ^77^ and the normalized Kratky plot was generated ^78^. Using the structure of sF13^WT^ (PDB: 9FHS) the experimental curve was compared to theoretical curve using CRYSOL ^76^. The SAXS statistics are provided in Tables S6 and S7.

### F13 transfection for Proximity Ligation Assay (PLA) and immunofluorescence (IF) staining

To perform the PLA experiment and IF staining of F13, HeLa cells were transfected with either a pcDNA 3.1 plasmids coding for F13^WT^ or F13^4MUT^ (N267D A288P A290V D294V), with an internal FLAG tag sequence (GGGDYKDDDDKGGG) inserted within residues D21 and N22. The use of an internal FLAG tag in F13 was necessary, as the N-terminus is buried into the membrane and the C-terminus is part of the dimeric interface. Thus, none of them were suitable for standard N- or C-terminal protein tagging. We selected the best region to insert the FLAG tag based on the sF13 dimer structure reported here. For this, we selected an exposed loop, away from the membrane interaction region and the dimerization interface.

For PLA and IF, 1.2 × 10^4^ HeLa cells/well were transfected in suspension using lipofectamine 2000 (Thermo Fisher Scientific) in a 96 well plate (μClear, Greiner Bio-One #655090) with 100 ng of DNA. In each well 50 μL of HeLa cells at 2.4 × 10^5^ cells/mL were mixed with 50 μL of transfection mix and 50 μL of DMSO or DMSO/tecovirimat, resulting in tecovirimat at a final concentration of 10 μM and DMSO at 0.1%. Cells were incubated 24 hours at 37 °C and 5% CO_2_, subsequently the cells were fixed with 4% PFA for 10 min.

### Proximity Ligation Assay (PLA)

PLA was performed using the Duolink PLA Fluoresence kit (Merck). In short, cells were permeabilized at room temperature (RT) for 3 min in PBS with Triton 0.1% and washed PBS. 40 μL of Duolink blocking solution was added to each well and the plate incubated at 37 °C for 1 hour. After blocking, cells were incubated at RT for 45 min with primary monoclonal mouse M2 (Merck) and rabbit D6W5B (Cell Signaling Technology) anti-FLAG antibodies (diluted in Duolink Antibody Diluent) at final concentration of 285 ng/mL. The wells were washed twice for 5 minutes at RT with Buffer A. 40 μL of PLA probe mix, containing PLA probe PLUS (anti-rabbit) and PLA probe MINUS (anti-mouse) were added to the wells following the manufacturer instructions. The plate was then incubated at 37 °C for 1 hour. After incubation, the wells were washed twice for 5 minutes with buffer A, then 40 μL of ligase mix was added to the samples following the manufacturer instructions. and incubated 30 minutes at 37 °C. Wells were washed twice for 5 minutes with buffer A at RT, subsequently, 40 μL of amplification mix (containing a polymerase) were added to each well, following the manufacturer instructions. The plate was then incubated for 100 minutes at 37 °C. Finally, PLA wells were washed once with buffer B for 10 minutes at RT, and a second time for 10 minutes at RT with buffer B supplemented with 1 μg/mL Hoechst 33342 nuclear staining (Invitrogen). Subsequently a final wash was performed with 0.01X buffer B for 1 minute at RT, and cells were then left in fresh PBS. The plates were imaged using an Opera Phenix Plus microscope (Revvity) at 20x. 49 images per well, covering over 90% of the well, were acquired.

### Immunofluorescence (IF) staining of F13

Immunofluorescence staining of F13 with rabbit and mouse anti-FLAG antibodies was performed in parallel to PLA, in the same plate. Cells were permeabilized at RT for 3 min in PBS with Triton 0.1% and washed PBS. 40 μL of Duolink blocking solution was added to each well and the plate incubated at 37 °C for 1 hour. Cells were incubated at RT for 45 min with primary monoclonal mouse M2 (Merck) and rabbit D6W5B (Cell Signaling Technology) anti-FLAG antibodies (diluted in Duolink Antibody Diluent) at final concentration of 285 ng/mL. The wells were washed twice for 5 minutes at RT with Buffer A. Wells were washed twice with PBS for 5 minutes at RT and Alexa FluorTM 488 goat anti-mouse antibody and Alexa FluorTM 488 goat anti-rabbit antibody (Invitrogen) (diluted in PBS, BSA 1%, Na Azide 0.1%) were added to the respective wells at a final concentration of 4 μg/mL. The plate was then incubated at 37 °C for 1 hour. Wells were washed once for 5 minutes at RT with PBS, and once with PBS supplemented by 1 μg/mL Hoechst 33342 nuclear staining (Invitrogen). Finally, cells were left in fresh PBS before imaging. The plates were imaged using an Opera Phenix Plus microscope (Revvity) at 10x. 21 images per well, covering over 90% of the well, were acquired.

### Viral Plaque Assay and analysis

6-well plates were seeded with BSC40 cells 24 hours before infection. Confluent BSC40 cells were infected with wild-type VACV-WR and rVACV mutants at a 10-fold dilution in DMEM with 2.5% FBS for 1 hour at 37°C. The infection medium was then removed, and a 0.5% methylcellulose in DMEM media overlay containing 10 μM TPOXX was added for 3 days at 37°C. Afterward, the overlay medium was removed, and the wells were fixed and stained with 1% crystal violet in 20% methanol for 20 minutes. The crystal violet was removed, wells were washed with PBS, and plates were imaged using a Cytation 7. Plaque counts and diameters were measured to determine titers (PFU/mL) and plaque sizes (μm) using program developed on the Cytation 7.

### Image analysis

PLA and IF images were analyzed using Signals Image Artist (Revvity). For PLA (Fig. S6), the PLA area (633 nm) was calculated using an intensity threshold. In parallel the number of cells was quantified using nuclear count on the Hoechst channel. The average PLA area per cell was calculated by dividing the total PLA area by the total number of nuclei per well. For quantification of the number of FLAG (F13) positive cells in each condition, first the number of cells was quantified using nuclear count on the Hoechst channel. Second, the number of nuclei positive for FLAG staining was calculated using an intensity threshold method on the nuclei region of interest (ROI). The percentage of FLAG+ cells was calculated by dividing the total number of FLAG+ nuclei by the total number of nuclei per well.

### Sequence analysis

For each viral species, sequences corresponding to the F13 coding sequence were extracted, and aligned using MAFFT v7.505. Alignments were manually curated for accuracy using Geneious Prime v2024.0.5, and sequences covering less than 70% of the coding sequence were discarded. After these steps, we obtained a dataset comprising F13 sequences from: 108 MPXV clade I, 8472 MPXV clade II, 81 VARV, 211 VACV, 13 ECTV, 98 CPXV, 11 CMLP, 1 BRPV, 6 Akhmeta_virus (AKMV), 2 Orthopoxvirus Abatino, 2 Skunkpox virus (SKPV), 2 Taterapox virus (TATV), 4 Raccoonpox virus (RCNV). Sequences were translated, and all variations to the consensus of each species were extracted.

## Supplementary Material

Supplement 1

## Data Availability

Atomic coordinates of the reported structures have been deposited in the Protein Data Bank under accession codes 9FHK, 9FHS, 9FI7, 9FJ1, 9FIZ, 9FJA, 9FJ0. All Molecular Dynamic Parameters (MDP), input topologies, coordinates, simulation control files and analysis scripts are provided. Files containing the sampled ΔH and ΔH/Δλ for ABFE are also included.
