# Supplementary material for: MECHANISMS OF TECOVIRIMAT ANTIVIRAL ACTIVITY AND POXVIRUS RESISTANCE
Source: Res Sq. 2024 Sep 23:rs.3.rs-5002222. Preprint. [Version 1] doi: 10.21203/rs.3.rs-5002222/v1 (PMC11469519; doi:10.21203/rs.3.rs-5002222/v1)
Supplement: Supplement 1 [file NIHPPRS5002222V1-supplement-1.pdf]

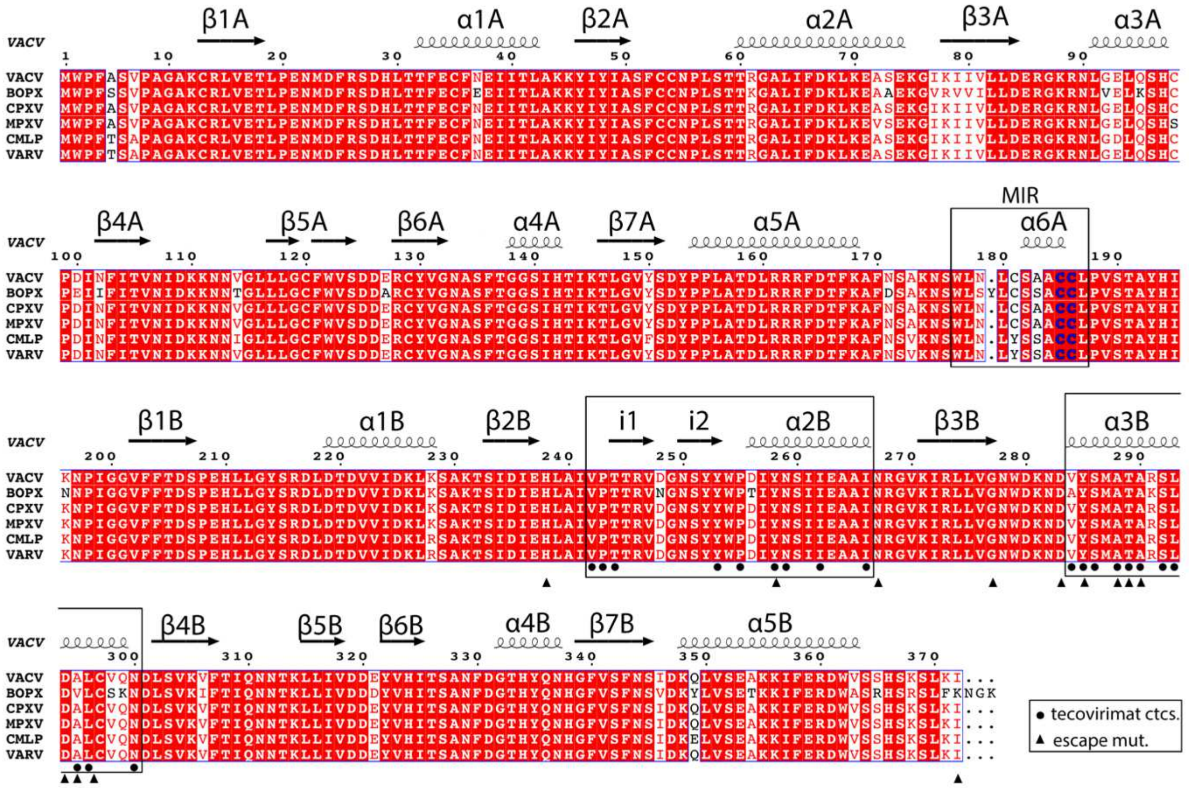

Figure S1. Multiple sequence alignment. Multiple sequence alignment from six representative OPXVs. The secondary elements are indicated at the top. Strictly conserved residues are highlighted in red. The palmitoylated cysteines are shown in blue and the membrane interacting region (MIR) framed and labeled. Tecovirimat contacts and positions of escape mutants are marked with black circles and triangles under the alignment, as indicated. The accession codes for the F13 proteins used in the alignment are: Borealpox virus (BOPX, QED21148.1), Camelpox virus (CMLP, A0A0K1LD56), Variola virus (VARV, AAA60785.1), Vaccinia virus (VACV, P04021), Monkeypox virus (MPXV, YP_010377040.1), Cowpox virus (CPXV, CAD90601.1). the alignment was performed using clustal omega [80] and the figure prepared with ESPRIPT [81].

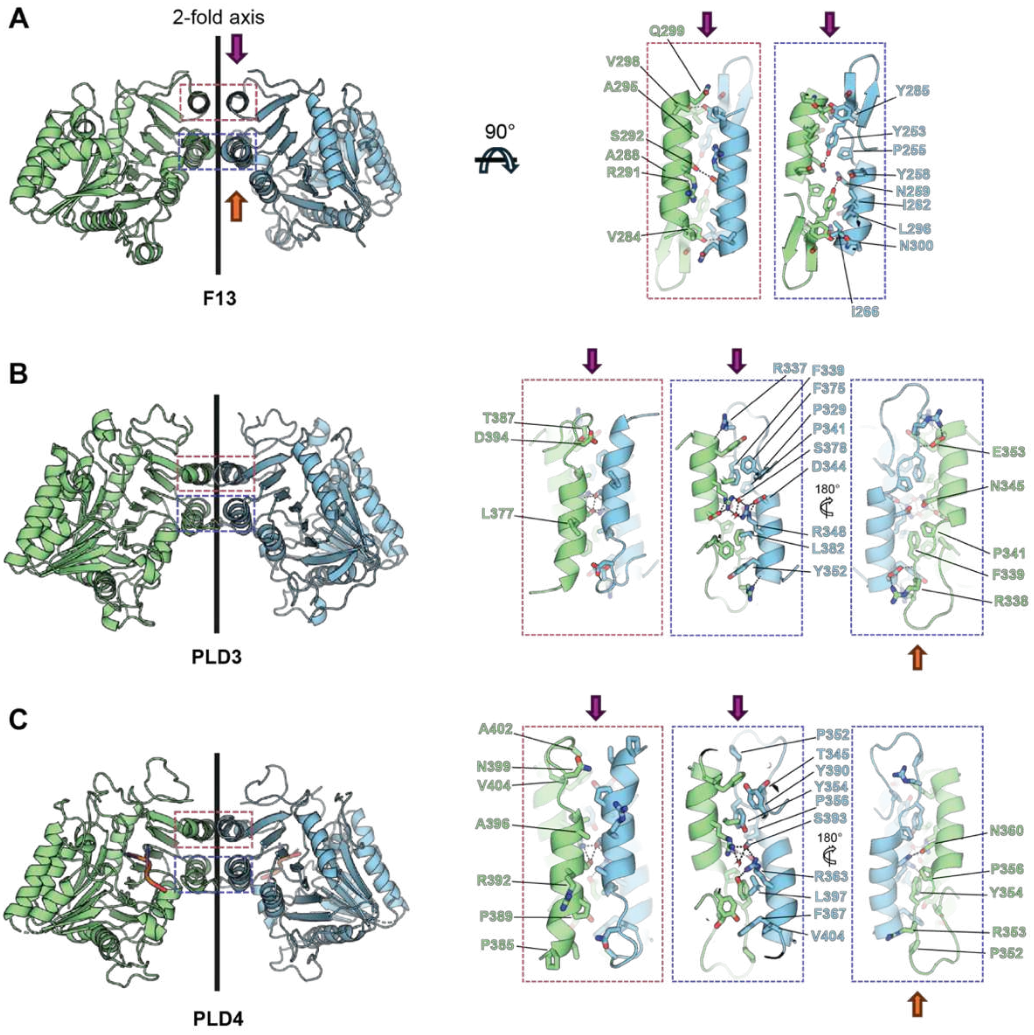

Figure S2. Contacts at the dimer interface. A) The left panel shows the crystal structure of the sF13 homodimer (PDB: 9FHS) represented in cartoon form. One protomer is colored blue, and the other is green. The 2-fold axis is indicated by a black line. The right panels provide two close-up views of the dimer interface, as indicated in the left panel. The purple and yellow arrows indicate a top view or a bottom view of the dimer interface, respectively, as shown in the left panel. The main residues contributing to the dimer interface (identified using the PDBePISA server) are depicted as sticks and labeled (green or blue). Polar contacts are shown as dashed black lines. B) Similar to A, the left panel shows the crystal structure of the PLD3 homodimer (PDB: 8V05), with protomers colored green and blue. The right panels are close-up views of the dimer interface. C) Similar to A, the left panel shows the crystal structure of the PLD4 homodimer (PDB: 8V08), and the right panels are close-up views of the dimer interface.

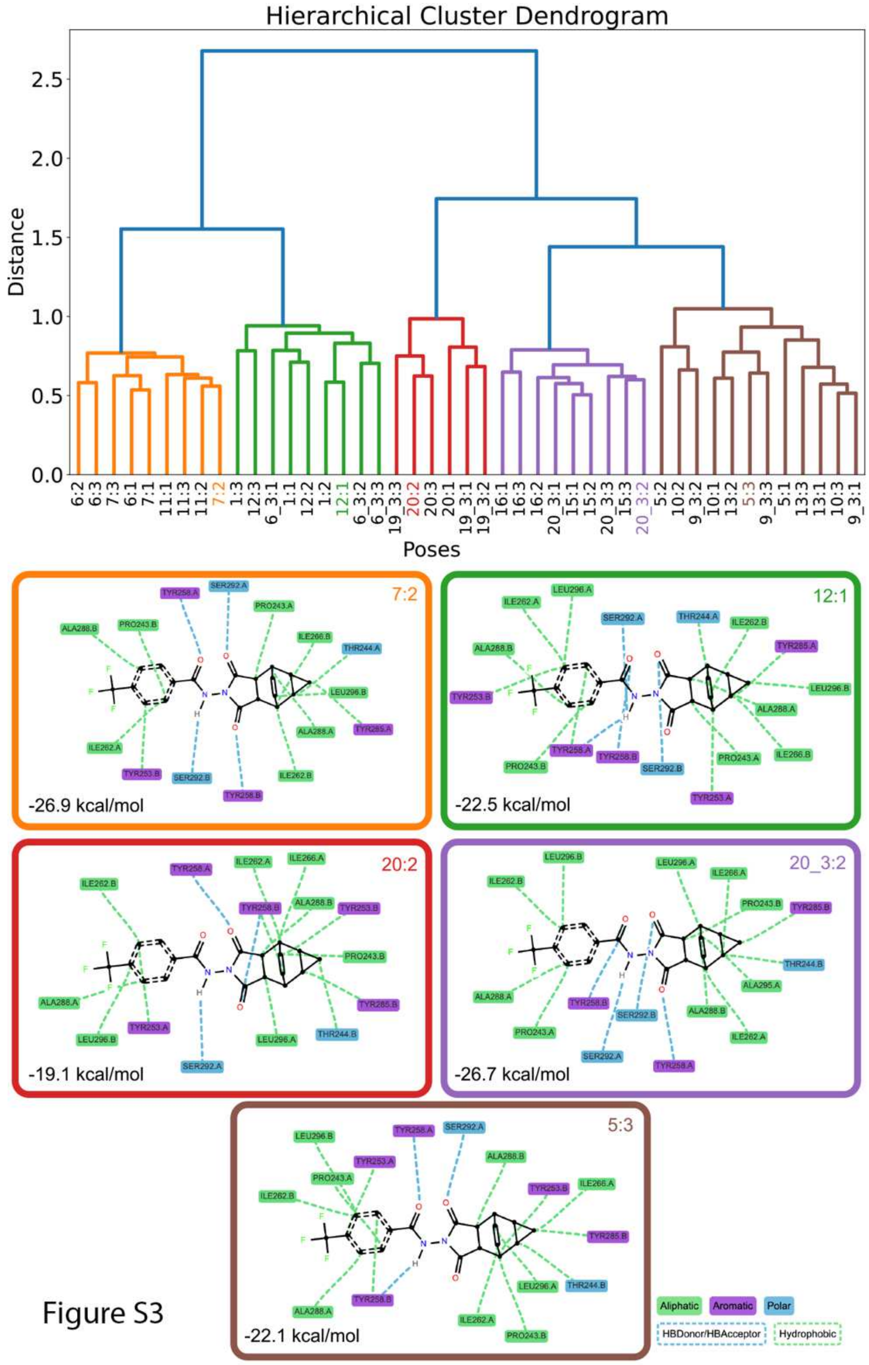

Figure S3

Figure S3. Cluster analysis of the 45 input structures for free energy perturbation simulations. Analysis of putative binding poses after equilibrating the 15 selected poses (x-label, first number) by three independent MD replicas (x-label, second number, see Methods). To reveal reoccurring protein-ligand interaction networks, a cluster analysis was performed. The x-axis represents the pose identification, and the y-axis indicates the distance between each pose in terms of their protein-ligand interaction network. The lowest energy pose of each cluster is highlighted, with its protein–ligand interaction profile and corresponding absolute

binding free energy. The analysis demonstrates (together with Table S4) that different protein-ligand interaction networks yield similar absolute binding free energies. The pose with the strongest binding affinity of -25.6 kcal/mol was used for further refinement against the crystallographic data, as reported in the PDB file. The Python library ProLIF [71] was used to generate the protein-ligand interaction network.

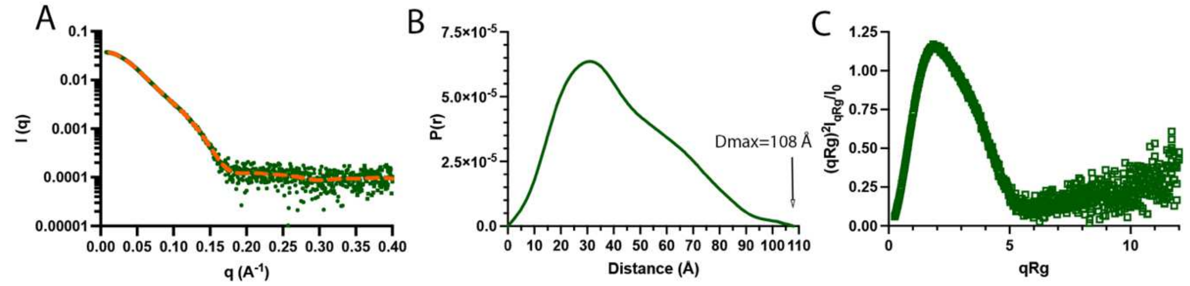

Figure S4. SAXS analysis of sF13/tecovirimat. A) Guinier plot showing the experimental scattering curve of the F13/tecovirimat complex in green, and the fitted curve used to generate the pair distance distribution in orange. B) Pair distance distribution function calculated using GNOM [77] used to obtain Dmax and Rg values. C) Dimensionless (normalized) Kratky plot showing the characteristic shape of a well-folded protein.

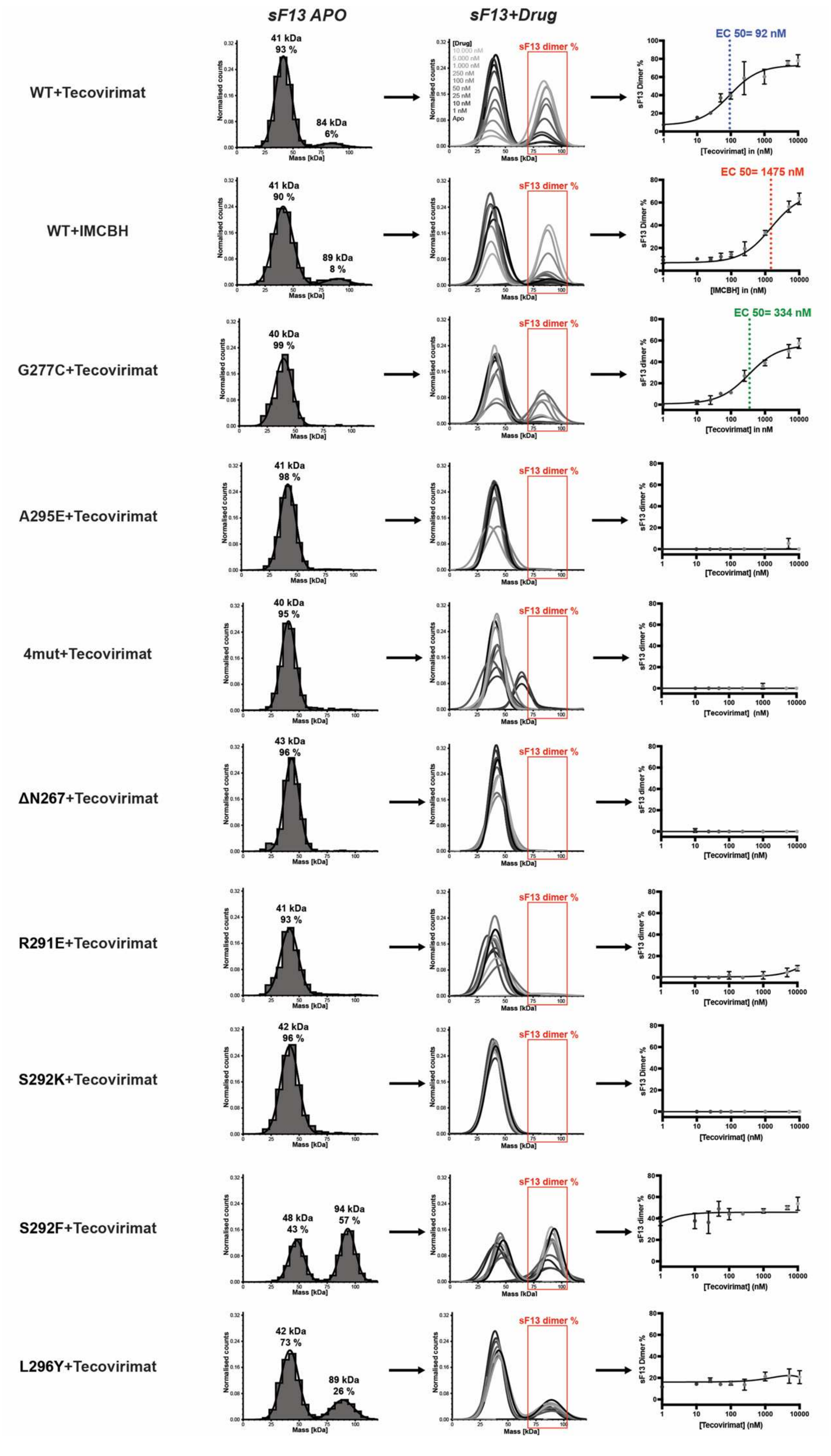
sF13 APO
sF13+Drug
WT+Tecovirimat
41 kDa 93 %
84 kDa 6%
sF13 dimer %
EC 50= 92 nM
WT+IMCBH
41 kDa 90 %
89 kDa 8 %
EC 50= 1475 nM
G277C+Tecovirimat
40 kDa 99 %
EC 50= 334 nM
A295E+Tecovirimat
41 kDa 98 %
4mut+Tecovirimat
40 kDa 95 %
ΔN267+Tecovirimat
43 kDa 96 %
R291E+Tecovirimat
41 kDa 93 %
S292K+Tecovirimat
42 kDa 96 %
S292F+Tecovirimat
48 kDa 43 %
94 kDa 57 %
L296Y+Tecovirimat
42 kDa 73 %
89 kDa 26 %
Normalised counts
Mass [kDa]
sF13 Dimer %
[Tecovirimat] in (nM)
[IMCBH] in (nM)
[Tecovirimat] (nM)

Figure S5. Mass photometry (MP) assay. Each row shows the pipeline used to determine EC50 values for sF13 wild-type and the different mutants, as indicated. The left column displays the mass distribution for sF13 at 25 nM without tecovirimat. The middle column shows mass distribution curves for sF13 with increasing drug concentrations, as indicated, highlighting in red the region used to calculate the percentage of dimers for the dose-response curve in the right column. The EC50 is derived from this dose-response curve. For clarity, the middle column presents a single representative experiment per drug concentration; however, the dose-response curve is based on the mean and standard deviation from three repeated experiments.

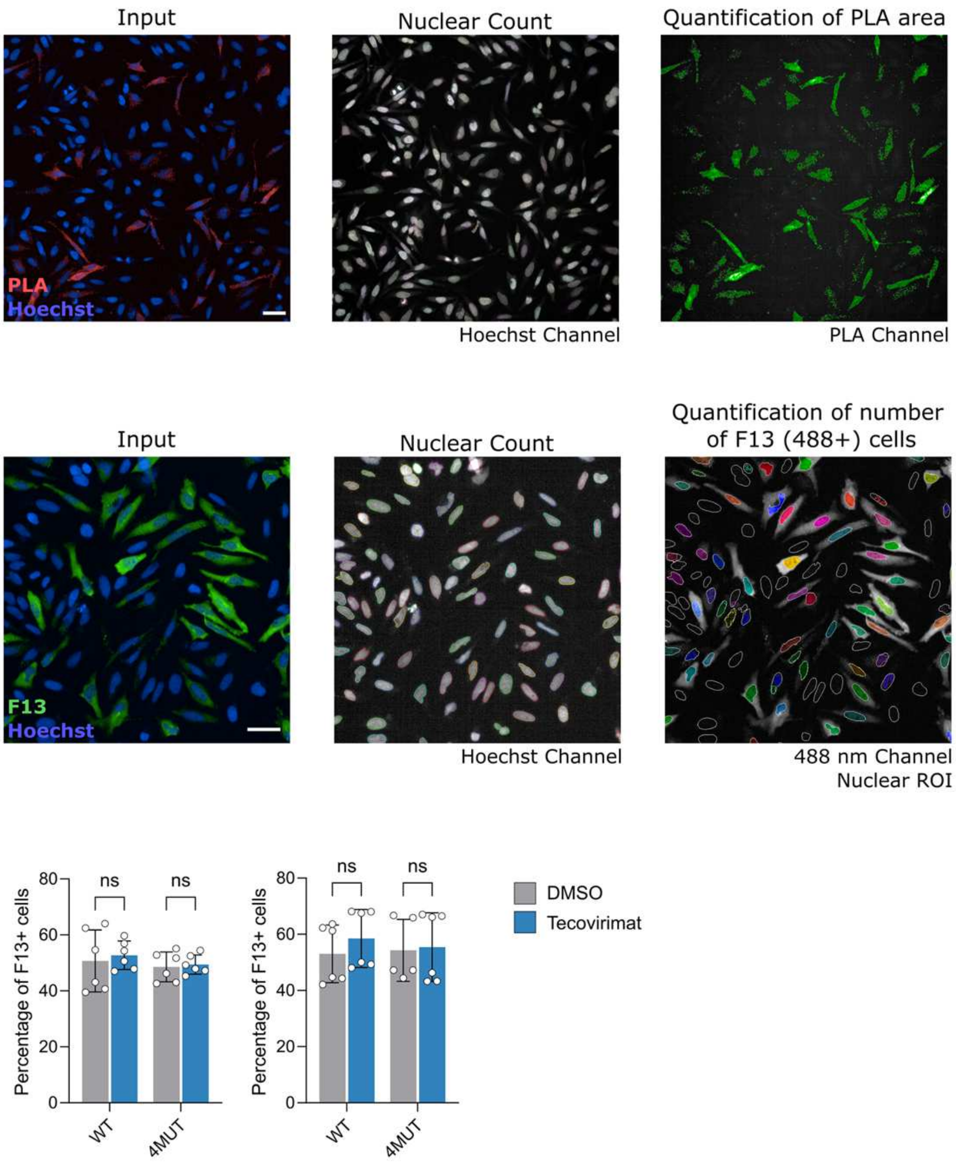

Figure S6. Image quantification methodology and percentage of F13-FLAG transfected cells. A) Quantification of the PLA signal area per cell. To measure the extent F13 dimerization, first, the number of Hoechst positive nuclei were automatically counted. Second, the PLA positive area was delimited and measured. Finally, the total PLA area was divided by the total number of nuclei. Scale bar: 50 µm. B) For percentage of transfection, total number of nuclei were automatically counted and delimited, then the total number of F13-FLAG positive nuclei were counted. Percentage of transfected cells was calculated by dividing the number of positive nuclei by the total number of nuclei. Scale bar: 50 µm. C) Quantification of the number of F13-FLAG positive cells for the indicated treatments. Left panel: anti-Mouse secondary antibody, right panel: anti-Rabbit secondary antibody. 7000 to 12000 cells were analysed per data point.

Data are mean±sd of two independent experiments performed in triplicat (n=6). Statistical analysis: Two-Way ANOVA. ns: non-significant.

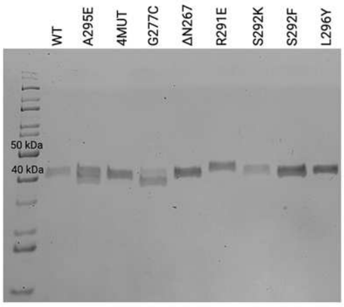

Figure S7. SDS-PAGE of the proteins used in this manuscript. Molecular weight markers are shown in the left lane. In the others 1 µg of sF13 wild-type and mutants has been loaded, as indicated.

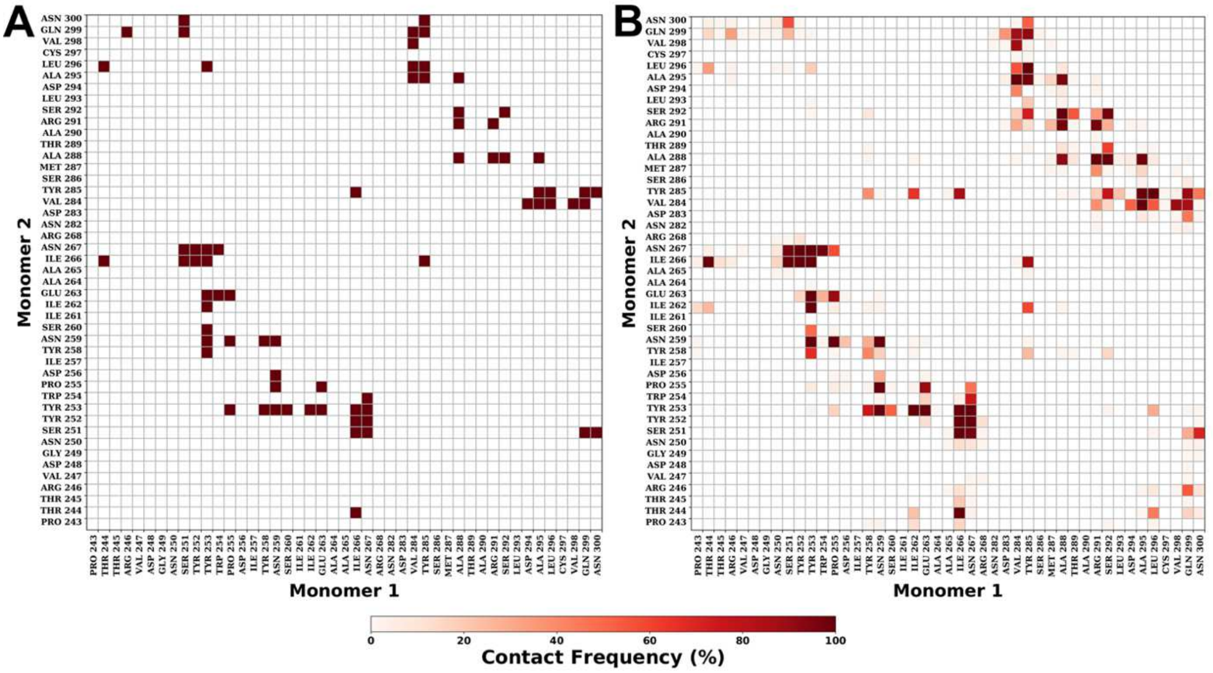

Figure S8. MD simulations of the F13 dimer on a lipid membrane. (A) Contact map showing interactions between the two monomers, calculated from the X-ray structure. (B) Contact map showing interactions between the two monomers, calculated from MD simulations by concatenating the last 300 ns from five repeats. The contact map highlights monomer-monomer interactions within 5 Å.

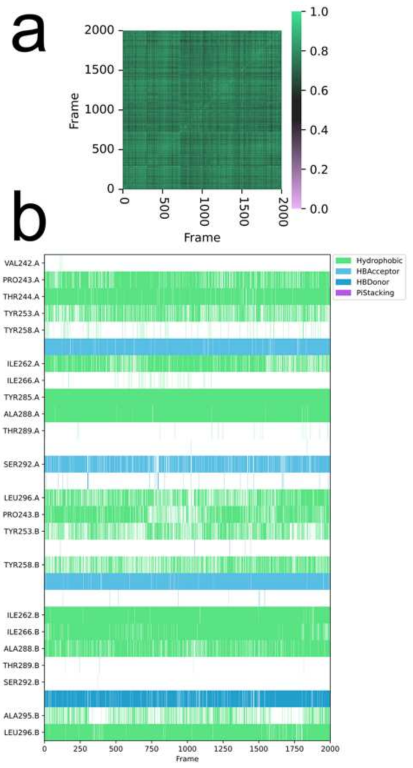

Figure S9. Protein-ligand interaction network analysis of the last 10 ns of the free simulation during the equilibration phase for pose 6-3. a) Tanimoto similarity matrix representing ligand-protein interactions across each frame of the MD trajectory. Values range from 0 to 1, where 1 indicates the highest similarity and 0 indicates the lowest. b) Barcode plot of interactions. Each horizontal line represents the presence of the corresponding interaction at a specific frame of the MD trajectory.

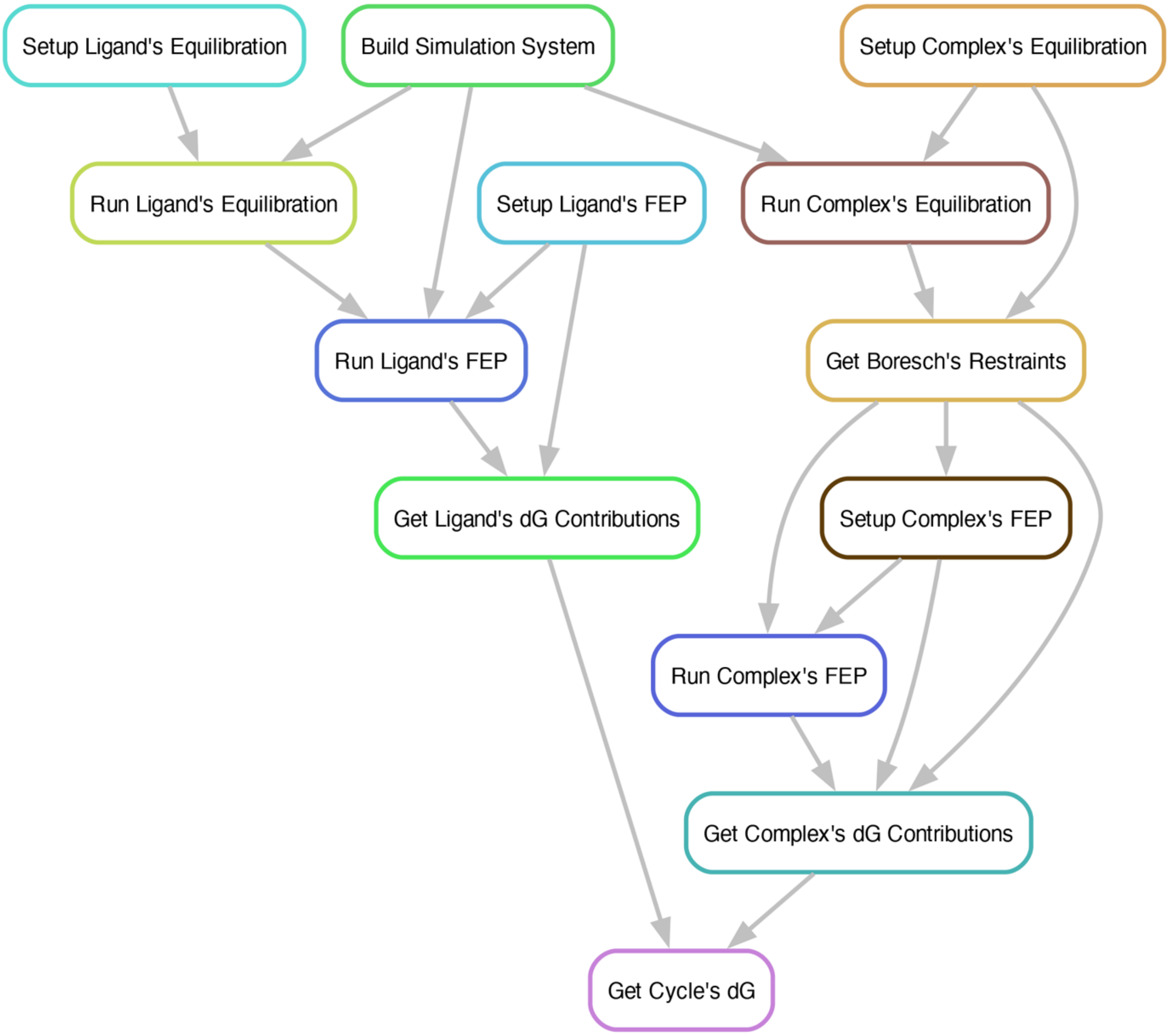

Figure S10. Protein

Table S1. Crystallographic statistics							
	F13 WT				F13 A295E		F13 G277C
	**APO**	**APO**	**Tecovirimat**	**IMCBH**	**APO**	**Tecovirimat**	**APO**
**PDB code:**	9FHK	9FHS	9FI7	9FJ1	9FIZ	9FJA	9FJ0
**Data collection:**							
Beamline	SOLEIL PX1	SOLEIL PX1	SOLEIL PX1	SOLEIL PX2	SOLEIL PX1	SOLEIL PX1	SOLEIL PX1
Space group	$P\ 1\ 2_1\ 1$	F 4 3 2	F 4 3 2	F 4 3 2	F 4 3 2	F 4 3 2	F 4 3 2
Unit cell parameters:							
a (Å)	52.35	280.36	281.09	282.80	280.98	281.72	281.05
b (Å)	94.11	280.36	281.09	282.80	280.98	281.72	281.05
c (Å)	73.19	280.36	281.09	282.80	280.98	281.72	281.05
β (º)	98.94						
Resolution (Å)	39.44 – 2.10	49.56 – 2.80	49.69 – 2.70	47.80 – 3.80	49.67 – 2.60	49.80 – 3.50	49.68 – 4.00
Last resolution bin (Å)	2.16 – 2.10	2.95 – 2.80	2.83 – 2.70	4.25 – 3.80	2.72 – 2.60	3.83 – 3.50	4.47 – 4.00
Total observations	287639 (24192)	1272138 (188317)	355137 (44348)	461213 (131203)	1718736 (212332)	693672 (169435)	657156 (186371)
Unique reflections	40522 (3297)	23822 (3397)	26783 (3453)	10019 (2745)	29770 (3553)	12621 (2936)	8519 (2335)
Completeness (%)	99.1 (99.1)	100 (100)	99.8 (98.8)	99.7 (99.0)	100 (100)	100 (100)	100 (100)
Redundancy	7.1 (7.3)	53.4 (55.4)	13.3 (12.8)	46.0 (47.8)	57.7 (59.8)	55.0 (57.7)	77.1 (79.8)
<I/s>	11.3 (1.0)	15.7 (0.3)	10.3 (0.5)	10.1 (1.0)	16.5 (0.2)	8.4 (-0.2)	11.9 (0.2)
R_{sym} (%)	7.3 (168)	23.5 (1438)	14.9 (412)	56.3 (560)	19.4 (2283)	39.5 (-6275)	27.7 (2510)
$CC_{1/2}$	99.8 (52.2)	99.9 (61.5)	99.8 (33.4)	99.7 (43.0)	99.9 (60.5)	99.9 (22.8)	100 (56.4)
B Wilson (Å^2)	54.21	100.87	89.21	158.11	73.64	117.88	189.54
**Refinement:**							
Resolution (Å)	39.44 – 2.10	49.56 – 2.80	49.69 – 2.70	47.80 – 3.80	49.67 – 2.60	49.80 – 3.50	49.68 – 4.00
Last resolution bin (Å)	2.15 – 2.10	2.95 – 2.80	2.79 – 2.70	4.19 – 3.80	2.69 – 2.60	3.85 – 3.50	4.58 – 4.00
Number of reflections	40483 (2693)	22527 (1895)	26706 (2430)	9986 (2264)	28632 (1723)	11873 (2197)	7954 (2096)
Nb of reflections used to calculate Rfree	2009 (166)	1141 (104)	1354 (116)	502 (123)	1396 (102)	589 (137)	401 (102)
B refinement	ISOTROPIC-TLS	ISOTROPIC-TLS	ISOTROPIC-TLS	ISOTROPIC-TLS	ISOTROPIC-TLS	ISOTROPIC-TLS	ISOTROPIC-TLS
Rfactor (%)	21.81 (35.98)	20.34 (78.59)	20.72 (62.71)	20.79 (30.51)	21.59 (83.18)	23.62 (51.20)	23.96 (50.25)
Rfree (%)	24.87 (39.06)	23.19 (85.59)	23.81 (56.60)	24.70 (36.61)	25.86 (73.99)	28.10 (51.87)	27.60 (56.58)
Number of atoms (Mean B value in A^2):							
sF13 (A/B)	2764 (65) / 2769 (68)	2884 (94)	2884 (85)	2884 (160)	2888 (88)	2888 (157)	2886 (254)
Citrate	-	13 (112)	13 (98)	13 (213)	13 (125)	-	-
Glycerol	-	72 (120)	48 (106)	12 (159)	78 (116)	18 (169)	6 (218)
Tecovirimat	-	-	27 (88)	-	-	27 (175)	-
IMCBH	-	-	-	20 (156)	-	-	-
Waters	70 (53)	58 (91)	32 (83)	-	59 (88)	14 (93)	-
Root mean square deviations:							
Bond lengths (Å)	0.002	0.002	0.044	0.002	0.002	0.044	0.002
Bond angles (º)	0.462	0.463	1.062	0.422	0.484	0.859	0.389
Ramachandran favored/outliers (%)	95.36 / 0.58	94.25 / 0.27	93.70 / 0.27	95.62 / 0.00	95.62 / 0.27	93.42 / 0.55	96.16 / 0.00

Table S1. Crystallographic statistics of all structures determined. Each column represents a different crystal structure data set, as indicated.

Lipid	Structure	Conc. (%)
POPC	16:0 - 18:1	11
PLPC	16:0 - 18:2	14
SAPC	18:0 - 20:4	20
PLPE	16:0 - 18:2	4
PSPE	16:0 - 18:0	8
SAPE	18:0 - 20:4	5
PSPI	16:0 - 18:0	2
POPI	16:0 - 18:1	5
SAPI	18:0 - 20:4	2
POPS	16:0 - 18:1	4
TSM	18:0 - 22:0	12
LPC16	16:0 - 0:0	5
CHOL	Cholesterol	8

Table S2. Lipid composition mimicking the mammalian Golgi membrane. Lipid tail structures and their concentrations are listed in the table

Table S3	Inhibition assay on MPXV		Mass Photometry assay	
**F13 residue substitutions**	**IC_{50} (μM)**	**Fold change[1]**	**EC_{50} (nM)**	**Fold change[2]**
K174N, N267D	12	720	-	-
H238Q	0.54–0.6	28–34	-	-
H238Q, N267D, A295E	24	1,400	-	-
H238Q, A288P, D294V, I372N	≈5.2	≈290	-	-
P243S, A288P, A290V	0.56	32	-	-
T245I, A290V	0.17	10	-	-
Y258C	18	1,000	-	-
N267D	10–11	570–630	-	-
N267D, A288P	1.2–16	71–900	-	-
N267D, A290V	2.0	110	-	-
N267D, D294V	12	680	-	-
N267D, A288P, A290V, D294V	>500	>29,000	>10,000	>110
N267D, A288P, A290V, A295E,L297ins	>500	>29,000	-	-
N267D, A288P, A290V, A295E,I372N	>500	>29,000	-	-
N267del	1.5–4.0	85–230	>10,000	>110
N267del, A290V	0.13	7.5	-	-
N267del, N267D, A295E	2.9–18	160–1,000	-	-
N267del, A288P, A295E	>500	>29,000	-	-
N267del, T289A, A295E	0.26	15	-	-
N267del, A290V, I372N	3.1	180	-	-
N267del, N267D, D294V, A295E	2.5	140	-	-
G277C	2.3-40	95-800	334	3.6
D283G	7.1–7.3	404–420	-	-
Y285H, I372N	0.045	2.6	-	-
A288P	0.5 to >500	29 to >29,000	-	-
A288P, I372N	>150	>8,600	-	-
A288P, A290V, D294V	0.66 to >500	38 to >29,000	-	-
A288P, A290V, L297ins	>500	>29,000	-	-
A288P, A290V, I372N	15	880	-	-
A288P, D294V, A295E	1.4	83	-	-
A288P, D294V, D301del	>500	>29,000	-	-
T289A	0.078–0.14	3.7–7.8	-	-
T289A, R291K	1.7	98	-	-
A290V	0.17–43	10–2,500	-	-
A290V, I372N	30–32	1,700–1,800	-	-
D294V	0.23–1.4	13–78	-	-
D294V, A295E	1	59	-	-
A295E	2.0–3.3	110–190	>10,000	>110
I372N	0.04–>150	2.3 to >8600	-	-

Table S3. List of tecovirimat resistance mutations detected on MPXV in U.S. during the 2022-2023 epidemic. We also included the escape mutant G277C, identified only in in vitro studies. MPXV, monkeypox virus. EC50, 50% effective concentration. "Ins" and "del" represents residues insertion and deletion respectively. IC50 fold changes were calculated based on a MPXV reference strain (clade IIa, U.S. 2003, IC50 = 0.0175 μM). EC50 fold change was calculated based on the results obtained in the mass photometry assay for F13 WT reported in this manuscript (EC50 = 93 nM).

Pose	Replica	MBAR				TI				Activate Boresch's restraints
		Delta G_bind	van der Waal	Coulomb	Release restraint	Delta G_bind	van der Waal	Coulomb	Release restraint	
1	1	-14,89	-22,542	2,013	-1,276	-14,82	-22,092	1,799	-1,438	6,914
	2	-17,66	-25,127	1,350	-1,116	-17,52	-24,864	1,320	-1,212	7,234
	3	-21,18	-29,620	2,537	-0,848	-21,12	-29,326	2,403	-0,955	6,753
10	1	-12,96	-20,602	2,485	-1,432	-12,59	-20,191	2,440	-1,425	6,587
	2	-18,53	-25,882	1,385	-0,742	-18,00	-25,411	1,467	-0,765	6,712
	3	-13,59	-23,426	4,270	-1,202	-12,84	-22,900	4,480	-1,191	6,772
11	1	-23,88	-32,042	1,964	-0,455	-22,51	-30,667	2,008	-0,496	6,649
	2	-24,95	-32,246	0,965	-0,474	-24,25	-31,527	0,974	-0,507	6,808
	3	-25,19	-30,307	-0,904	-0,790	-24,79	-30,011	-0,777	-0,815	6,813
12	1	-22,45	-28,858	0,795	-1,142	-21,39	-27,893	0,894	-1,148	6,759
	2	-16,54	-25,032	2,554	-0,787	-16,43	-24,869	2,565	-0,847	6,725
	3	-15,09	-23,324	2,349	-0,834	-14,58	-22,804	2,401	-0,897	6,720
13	1	-15,12	-24,264	3,304	-1,036	-14,75	-23,782	3,257	-1,100	6,878
	2	-18,25	-26,592	2,926	-1,315	-17,76	-26,234	3,026	-1,286	6,729
	3	-16,75	-25,653	3,478	-1,396	-16,56	-25,390	3,455	-1,444	6,823
15	1	-22,60	-29,654	0,971	-0,604	-22,22	-29,257	0,978	-0,633	6,691
	2	-16,34	-22,528	0,185	-0,745	-15,74	-22,052	0,321	-0,761	6,749
	3	-19,25	-24,537	-0,831	-0,642	-18,84	-24,181	-0,759	-0,668	6,764
16	1	-21,44	-26,627	-0,533	-0,897	-20,77	-25,962	-0,489	-0,936	6,620
	2	-16,48	-22,323	-0,471	-0,562	-16,18	-22,236	-0,233	-0,592	6,878
	3	-24,54	-30,730	0,055	-0,494	-24,17	-30,363	0,079	-0,520	6,632
19_3	1	-16,39	-23,041	0,940	-1,040	-16,24	-22,659	0,976	-1,314	6,753
	2	-14,88	-23,554	3,082	-0,897	-14,65	-23,210	2,990	-0,923	6,489
	3	-16,64	-23,874	1,030	-0,584	-16,38	-23,488	0,927	-0,608	6,793
20	1	-12,15	-19,279	1,483	-0,951	-11,96	-19,134	1,511	-0,930	6,594
	2	-19,05	-26,397	1,088	-0,575	-18,74	-26,044	1,069	-0,598	6,835
	3	-18,81	-25,450	0,245	-0,485	-17,94	-24,640	0,412	-0,599	6,885
20_3	1	-20,94	-27,894	1,217	-1,007	-20,64	-27,503	1,131	-1,014	6,747
	2	-26,73	-32,534	-0,536	-0,670	-26,24	-32,148	-0,377	-0,721	7,009
	3	-23,19	-29,985	0,341	-0,684	-21,99	-28,745	0,347	-0,731	7,138
5	1	-18,24	-25,712	3,056	-2,387	-18,70	-25,397	2,972	-3,081	6,802
	2	-14,86	-23,050	2,557	-0,967	-13,98	-22,260	2,655	-0,974	6,598
	3	-22,11	-29,452	1,610	-1,005	-22,09	-29,313	1,678	-1,192	6,735
6	1	-22,74	-28,184	-0,485	-0,663	-22,18	-27,669	-0,414	-0,698	6,596
	2	-18,38	-24,804	0,278	-0,529	-17,95	-24,392	0,332	-0,566	6,677
	3	-25,77	-33,454	1,623	-0,645	-25,53	-33,218	1,647	-0,664	6,700
6_3	1	-22,34	-28,062	-0,464	-0,569	-22,34	-28,029	-0,443	-0,625	6,753
	2	-18,73	-28,855	4,516	-1,094	-18,29	-28,489	4,641	-1,152	6,707
	3	-19,70	-27,439	2,493	-1,488	-19,64	-27,113	2,482	-1,744	6,735
7	1	-20,93	-27,579	0,259	-0,515	-20,40	-27,329	0,550	-0,520	6,901
	2	-26,94	-33,223	-0,137	-0,520	-26,84	-33,122	-0,106	-0,554	6,944
	3	-19,04	-24,425	0,086	-1,358	-18,18	-23,470	0,117	-1,484	6,656
9_3	1	-17,05	-25,236	1,944	-0,568	-16,44	-24,816	2,167	-0,602	6,807
	2	-16,92	-25,526	2,587	-0,713	-16,38	-25,012	2,619	-0,721	6,733
	3	-18,72	-27,425	2,745	-0,768	-18,25	-26,979	2,812	-0,811	6,730

Table S4. Binding free energy estimation by MBAR and TI with its corresponding energy contributions for all simulations. Units are in kcal/mol.

Pose	Replica	MBAR				TI				Activate Bore
		Delta G_bind	van der Waal	Coulomb	elease restrain	Delta G_bind	van der Waal	Coulomb	elease restrain	
A	1	-4,41	-10,692	1,724	-1,964	-4,83	-10,445	1,752	-2,658	6,524
	2	-4,30	-9,457	0,146	-1,738	-3,95	-9,289	0,397	-1,811	6,751
	3	-3,05	-9,044	0,832	-1,578	-2,76	-8,894	1,021	-1,626	6,741
B	1	-5,16	-10,637	-0,541	-0,813	-4,82	-10,288	-0,549	-0,817	6,833
	2	-6,26	-11,127	-0,846	-0,638	-6,07	-10,974	-0,793	-0,646	6,347
	3	-6,27	-11,223	-1,112	-0,871	-6,46	-10,871	-1,080	-1,445	6,938

Table S5. Binding free energy estimation by MBAR and TI with its corresponding energy contributions for pose 6-3 on the individual monomers. Units are in kcal/mol.

Data collection parameters

Instrument	Beamline SWING (synchrotron SOLEIL)
Detector	CCD-based AVIEX
Beam geometry	0.8 mm x 0.15 mm
Wavelength [Å]	1.0
q-range [$Å^{-1}$]	$0.0064 < q < 0.40$
Exposure time [s]	1.5
Temperature [K]	288

Structural parameters

I(0) Guinier [cm^{-1}]	0.039
R_g Guinier [Å][a]	32.1
I(0) *P*(r) [cm^{-1}]	0.039
R_g *P*(r) [Å]	32.9
D_{max} [Å]	108

Molecular mass determination

	**Estimated MM [kDa]**
$MM_{sequence}$[b]	88.6
MM_{SAXS} QR [c]	83.6
MM_{SAXS} QR [d]	76.3

[a] R_g obtained with the Guinier approximation in the range $qR_g < 1.3$

[b] The calculated masses were derived from the sequence

[c] Molecular mass obtained from the I(q) curve (q_{max} = 0.249 $Å^{-1}$) using the MoW3 program, available at http://saxs.ifsc.usp.br

[d] Molecular mass obtained from bayesian interference analysis

Table S6. SAXS data collection and scattering derived parameters.

	R_g [Å]	D_{max} [Å]	Chi^2	S [S]	f/f_0
**Experimental value**					
F13/Tecovirimat complex	32.1	108	ND	4.7 ± 0.2	1.45
**Coral models Calculated values**					
F13 monomer	21.3	72	234	ND	ND
F13 dimer	32.3	117	1.58	ND	ND

Table S7. SAXS data. A) Experimental values were measured by SAXS or AUC. Theoretical curves were generated by Crysol from monomeric and dimeric structure and Chi2 value of the model were calculated by comparison to the experimental SAXS curve. Rg, Dmax were determined with HYDROPRO for all structure with an average size for each bead of 3.24 Å.
